# Counter Anion Effect on the Photophysical Properties of Emissive Indolizine-Cyanine Dyes in Solution and Solid State

**DOI:** 10.3390/molecules23123051

**Published:** 2018-11-22

**Authors:** Jacqueline N. Gayton, Shane Autry, Ryan C. Fortenberry, Nathan I. Hammer, Jared H. Delcamp

**Affiliations:** Department of Chemistry and Biochemistry, University of Mississippi, University, MS 38677, USA; jgayton@go.olemiss.edu (J.N.G.); saautry@go.olemiss.edu (S.A.); r410@olemiss.edu (R.C.F.); nhammer@olemiss.edu (N.I.H.)

**Keywords:** NIR emissive materials, stokes shift, indolizine cyanine, solid-state emission, optical materials

## Abstract

Near-infrared emissive materials with tunable Stokes shifts and solid-state emissions are needed for several active research areas and applications. To aid in addressing this need, a series of indolizine-cyanine compounds varying only the anions based on size, dipole, and hydrophilicity were prepared. The effect of the non-covalently bound anions on the absorption and emission properties of identical π-system indolizine-cyanine compounds were measured in solution and as thin films. Interestingly, the anion choice has a significant influence on the Stokes shift and molar absorptivities of the dyes in solution. In the solid-state, the anion choice was found to have an effect on the formation of aggregate states with higher energy absorptions than the parent monomer compound. The dyes were found to be emissive in the NIR region, with emissions peaking at near 900 nm for specific solvent and anion selections.

## 1. Introduction

Near infrared (NIR) emissive materials in both solution and solid-state are in high demand, with applications in biological imaging and optoelectronics [1,2,3,4,5,6,7,8,9,10,11]. For non-invasive biological imaging an increased Stokes shift (the energetic difference between the absorption and emission maxima) is desirable to increase image resolution [12,13]. Recently, we reported that Stokes shifts can be controlled through the molecular engineering of π-conjugated materials by the introduction of strategic dye steric elements and ground state ring strains [14,15,16]. Presumably, non-covalently bound counter ion selection with charged π-systems could also be used to tune reorganization energies by altering the chromophore environment. The counter ion size would also be expected to have a significant impact on film morphologies and therefore influence solid-state properties.

**Figure 1:**
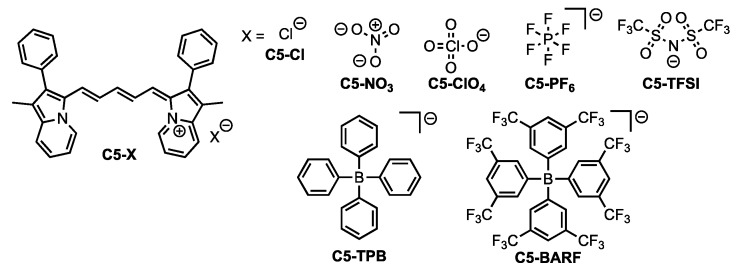
Dye **C5** with counter anions studied in this work.

Cyanine dyes are a common class of NIR emissive materials known for their intense molar absorptivities (ε) and high molecular brightness (MB = ε x Φ, where Φ is the quantum yield defined as number of photons emitted per number of photons absorbed) in the NIR region [7]. Cyanine dyes often suffer from small Stokes shifts and prior efforts have focused on increasing reorganization energies without loss of emission [17,18]. Recently, we designed a series of cyanine dyes utilizing an indolizine donor in place of the common indoline donor [19]. A steric-induced deviation from typical bond angles along the cyanine backbone in the ground state geometry was found to reorganize to more ideal bond angles in the excited state. The reorganization energy in changing from the ground state to the excited state geometry gave an increased Stokes shift (up to 60 nm) relative to common indoline cyanine Stokes shifts of ≤20 nm [17,20,21,22]. Thus, indolizine cyanine dyes are good candidates to evaluate the effects of non-covalent anions on the dye photophysical properties since the π-conjugated system is positively charged and significant reorganization energies can be adopted. Anion choice has been shown to have substantial effect on dye optical properties previously in literature [23,24,25,26,27,28,29]. To analyze the effects of anion selection on the recently reported indolizine cyanine dye C5 [19], seven anions with varying size and charge distributions were targeted (Figure 1). Chloride (Cl^−^), perchlorate (ClO_4_^−^), hexafluorophosphate (PF_6_^−^), and nitrate (NO_3_^−^) were selected as smaller-sized ions. Trifluoromethanesulfonimide (TFSI), tetrakis[3,5-bis(trifluoromethyl)phenyl]borate (BARF), and tetraphenylborate (TPB) were selected as the largest anions studied. By systematic study of these anions with C5, the effects of anion properties on the conjugated system photophysical properties can be examined in both solution and the solid-state.

## 2. Results and Discussion

First, the anions were analyzed computationally to determine ion volume, electrostatic surface potentials, and Mulliken population charges with the Gaussian 09 program [30] via B3LYP/6-31+G(*d*) [31,32,33] in a fashion similar to that used for determination of charge densities in common ions [34]. Comparing ion sizes by volume, the following order of ions was established from smallest to largest: NO_3_^−^ ≈ Cl^−^ < ClO_4_^−^ < PF_6_^−^ < TFSI << TPB << BARF (Table 1). BARF is nearly double the size of the next-largest ion, TPB, which is nearly double the size of TFSI. Comparing the range of anion sizes, Cl and NO_3_ are more than 10 times smaller than BARF. This vast difference in size of the selected ions allows for the probing of anion size effects on dye properties, which would presumably be heavily affected in the solid state, where the anion size plays a significant role in the determination of dye–dye interaction distances.

**Figure 2:**
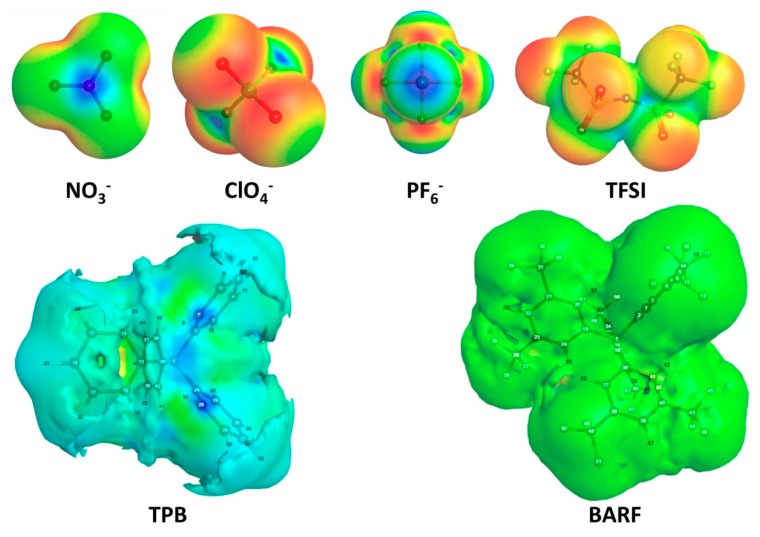
Electrostatic surface potentials of anions.

The electrostatic surface potential maps for each of the anions are plotted with the WebMO graphical user interface [35] in Figure 2 to see the outer atom charges, which interact most strongly with the cationic dye C5. Among the polyatomic anions studied NO_3_^−^, ClO_4_^−^, PF_6_^−^, and TFSI all show significant negative charge build up on the outer periphery of the anions. Mulliken population charges show the largest accumulation of negative charge on the outer atoms of the anion for PF_6_^−^ with a charge of −0.734 on each of the F atoms (Tables S1–S6). The trend then proceeds in the following order from most negative charge on peripheral atoms to least: PF_6_^−^ > ClO_4_^−^ > TFSI > NO_3_^−^ >BARF> TPB. TPB is unique among the anions in that the outermost atoms (hydrogens) show a build-up of positive charge around the periphery of this anion. This is clearly displayed on the electrostatic surface potential map where the surface of the TPB anion is nearly completely blue indicating build-up of positive charge (Figure 2). Given the outer positive charge surrounding the TPB anion, it is expected to be very weakly attracted to the cationic C5 dye relative to the other anions with negative charge build-up on the surface.

**Figure 3:**
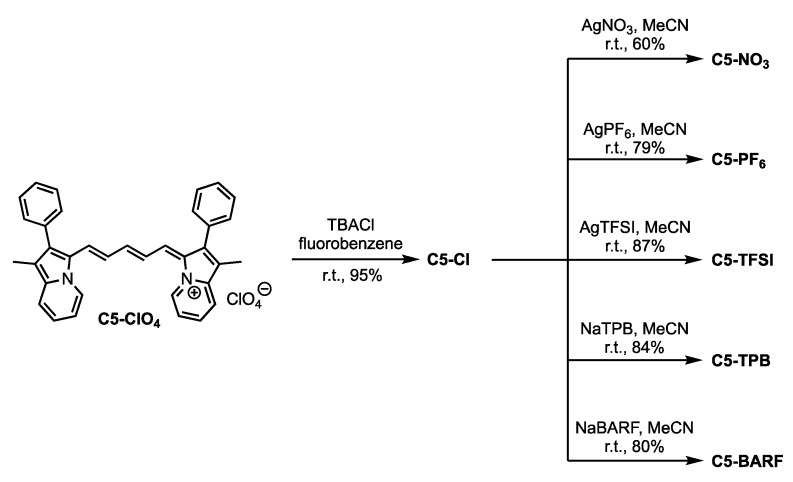
Synthetic route to the **C5** anion varied compounds.

The target dyes were synthesized beginning from previously reported C5-ClO_4_ [19]. C5-Cl could be accessed from C5-ClO_4_ in high yield (95%) via a salt metathesis with tetrabutylammonium chloride (TBACl) in fluorobenzene (Figure 3). The significant solubility difference in C5-ClO_4_ (fluorobenzene soluble) and C5-Cl (fluorobenzene insoluble) suggests a successful anion swap. C5-Cl was further purified with column chromatography where the unique R_f_ of C5-Cl relative to C5-ClO_4_ further confirms ion exchange. The complete anion exchange is verified by metathesis reactions with C5-Cl and sodium salts of TPB and BARF which show quantitative anion exchange via ^1^H NMR. If ClO_4_^−^ were remaining, equal molar ratios of TBP or BARF to C5 would not be observed. C5-Cl serves as a lynch pin intermediate in allowing access to C5-NO_3_, C5-PF_6_, C5-TFSI, C5-TPB, and C5-BARF from silver and sodium salts of the anions via metathesis reactions. The presence and quantification of the TPB and BARF anion could be confirmed by ^1^H NMR. The presence of PF_6_^−^, TFSI and BARF were confirmed by ^19^F NMR and the drastic solubility differences between C5-Cl and C5-PF_6_ or C5-TFSI suggests complete metathesis reactions. All dyes were purified via column chromatography after metathesis reactions to remove any silver or sodium salts followed by filtration of the dyes through celite to remove any residual solid phase particles remaining in the sample due to the use of methanol as an elution solvent in most cases.

**Figure 4:**
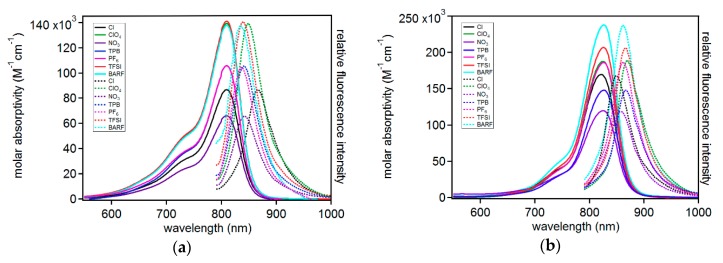
Molar absorptivity (solid lines) and emission intensity (dotted lines) for each dye in (**a**) acetonitrile and (**b**) dichloromethane.

With the target C5-anion varied compounds in hand, molar absorptivity and emission spectrum were measured for each dye in a polar aprotic solvent (acetonitrile, MeCN) and a low-polarity aprotic solvent (dichloromethane, DCM; Figure 4, Figures S10 and S11; Table 2). The absorption curve shapes show sharp transitions at 810 nm in MeCN or 825 nm in DCM. The sharp transition shows a peak ½ width of only 75 nm (0.15 eV, 1176 cm^−1^). The narrow transition is indicative of a π-π* transition as is commonly observed for many symmetric indoline cyanine dyes [36] rather than a charge transfer (CT) transition, which is typically much broader in this spectral region (>150 nm, >0.46 eV, >3735 cm^-1^) [15,16]. Additionally, the curve shape for these dyes shows a high energy shoulder which is thought to be a characteristic vibronic feature of many indoline-cyanine dyes undergoing low energy π-π* transitions [37]. Anion selection has a dramatic effect on molar absorptivity in MeCN with a range of 141,000 to 66,000 M^-1^cm^-1^ where ε is increasing in the following order: NO_3_^−^ < Cl^-^ < PF_6_^−^ = TPB < BARF < ClO_4_^−^ < TFSI when an absorbance of 1 is reached. We note that the MeCN ε measurements via serial dilution reveal a positive deviation from the Beer Lambert Law, even at very low concentrations, while DCM shows linear absorbance versus concentrations plots even to high absorbances near 3 in some cases (Figures S12–S23). An example spectra set is provided at varying concentrations where new features are observed as concentrations change in MeCN (Figure S24). The origin of this positive deviation is not obvious as the samples appear dissolved to the naked eye, although the possibility of non-observed solution aggregates exists. This non-linear behavior is observed even at low absorbances near the detection limit of the spectrometer used in these studies. The anion trend does not track with either simplistic analysis of anion size or charge localization and we note these factors as well as others such as dispersion forces may be in competition. The smallest anion and the largest anion had the highest molar absorptivities, and the order of anion charge localization is not mirrored in the molar absorptivity measurements as found via computational analysis. Significant dispersion forces with small ions may promote higher molar absorptivities and larger anions promote higher molar absorptivities through increased intermolecular spacing leading to two independent strong effects which does not allow for a definitive simple trend analysis. No significant change in molar absorptivity maximum (λ_max_^abs^) is observed with all compounds having a maximum absorbance at 810 nm in MeCN. In DCM, an even more dramatic molar absorptivity change is seen with anion choice ranging from 120,000 to 238,000 M^-1^cm^-1^. The anion trend in DCM is as follows: NO_3_^−^ < TPB < Cl^−^ < PF_6_ < ClO_4_^−^ < TFSI < BARF. This trend tracks reasonably well with anion size suggesting the influence of larger anions on π-system spacing plays a dominant role. The three largest anions are in order of size with higher molar absorptivities than the remaining anions. The difference in MeCN and DCM can be rationalized as the less polar solvent (DCM) giving a stronger contact ion pair between the anions and cations than the more polar MeCN. This tighter ion pair shows a very dramatic ε change relative to the better solvated ions in MeCN. The anions in DCM again had no significant effect on λ_max_^abs^ with all compounds absorbing at ~825 nm.

The emissive properties of the dyes were also analyzed in both MeCN and DCM (Table 2). In MeCN, C5-PF_6_ and C5-BARF have the highest energy emission and C5-Cl has the lowest energy emission, with the remaining anions following this energy trend: PF_6_^−^ ≈ BARF > TFSI > TPB > NO_3_^−^ > ClO_4_^−^ >> Cl^−^. Since all the compounds have the same absorption energy, the Stokes shift values are directly correlated with the observed emission energy changes. C5-Cl has a 56 nm (0.10 eV, 798 cm^-1^) Stokes shift which is the largest observed in the series. The smallest Stokes shift in MeCN was with C5-PF_6_, at half the energy of the chloride derivative (0.05 eV, 384 cm^-1^). This change in energy of emission illustrates a larger geometry reorganization of the excited state in the presence of chloride relative to PF_6_. In this polar solvent the localized anion has a larger effect on the geometry of the excited state, which may be due to a strong contact ion pairing being needed to keep the anion near the dye when dissolved in MeCN. The remaining anions are likely more readily dispersed in MeCN. Interestingly, in the less polar DCM as solvent, the effect of the anions on emission energy changes substantially with a new order of increasing Stokes shift energies: Cl^−^ > NO_3_^−^ > BARF > PF_6_^−^ > TFSI > TPB > ClO_4_^−^. In this solvent, the chloride ion has the smallest Stokes shift (0.05 eV, 402 cm^-1^) with ClO_4_^−^ having nearly a twice as large of a Stokes shift at 0.09 eV (600 cm^-1^). Thus, the effect of the anions on emission energy and absorption intensity varies significantly with solvent, as well as anion, selection. These molar absorptivity and emission energy results in two different solvents with each set of anions highlights the importance of carefully comparing dyes in literature under identical conditions since both solvent and anion choice are non-innocent.

In most cases, the dyes were too weakly emissive to accurately measure a quantum yield of emission. This is expected, based on the Energy Gap Rule, which predicts that as lower-energy light is absorbed and emitted, thermal pathways start to dominate excited state energy loss mechanisms rather than photon emission [38,39,40,41]. However, despite the indolizine cyanines absorbing and emitting light significantly further into the NIR region than traditional indoline cyanines, several compounds were found to have quantum yields measured in excess of 1%. C5-ClO_4_ was found to be emissive in both MeCN and DCM at 1.2 and 2.2% Φ, respectively. C5-ClO_4_ was the only compound found have a >1% quantum yield in DCM. C5-TFSI and C5-TPB were both emissive at 1.1 and 1.2% Φ, respectively, in MeCN. These results suggest that ClO_4_^−^, TPB, and TFSI function most effectively in reducing thermal energy losses from the excited state dye. By analyzing molecular brightness of the compounds, both the intense absorption strength and quantum yield can be accounted for to generate a number valuable to applications of emissive materials where both components are critical such as biological imaging. The highest MB (4,100) was observed for C5-ClO_4_ in DCM due to a high Φ and medium molar absorptivity relative to the other compounds in DCM. The highest MB confirmed in MeCN is also with C5-ClO_4_ and is significantly lower than that observed in DCM at 1,700. This lower MB is due to both a lower molar absorptivity in MeCN than DCM and a much lower quantum yield in MeCN. It should be noted that since the quantum yields are only reliably reported as <1% in several instances, the exact MB value is not known for many of the of the compounds and only a possible upper limit can be defined in Table 2.

**Figure 5:**
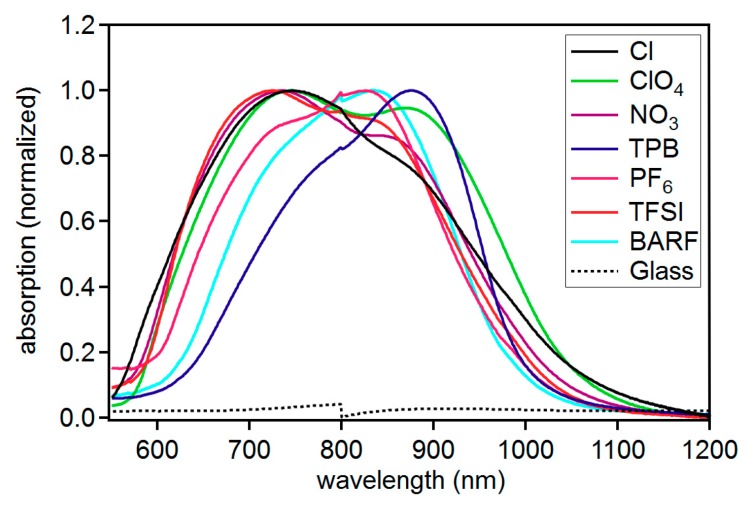
Solid-state absorption spectra for C5-X compounds.

In addition to solution studies, the solid-state absorption and emission properties were examined for each compound to determine the influence of anions on solid-state photophysical properties (Figure 5). Thin films of each dye were prepared by first dissolving each dye in a 1:1 mixture of acetonitrile:chlorobenzene (~0.01 M dye solution) and spin coating a glass slide. To ensure the absorption spectrum were resulting from the dye and not the substrate, a non-normalized spectrum set is provided with the blank glass slide absorption which shows a clear difference (Figure S25). Additionally, films were made via spin coating from different solution concentrations which shows a significant change in absorption intensity and on a different surface (fluorine doped tin oxide (FTO)) with a different glass supplier (Figures S26–S28). These control experiments suggest the absorption features are a result of the dye and not the substrate. The anion was found to have a significant effect on the absorption curve shape. C5-TPB was found to uniquely have a curve shape most resembling the solution spectrum with a large low energy feature and a presumably vibronic higher energy shoulder. The ratio of the shoulder feature to low energy transition differs significant from the solution measurements with a roughly 0.8:1 ratio in the solid state for C5-TPB and a 0.3:1 ratio in solution. Additionally, the absorption energy maximum has shifted significantly to 878 nm from solution measurements at 810 or 826 nm (Table 3). A host of reasons exist to explain this red-shift of film absorption relative to solution with examples such as a significant change in the π-conjugated system geometry in the ground state in the solid state relative to solution, excimer formation, π-stacking, and various aggregation phenomena.

The remaining anions show an increase in the prominence of the higher energy shoulder region ranging from a near 1:1 ratio with the low energy feature for BARF and ClO_4_^−^ to favoring of the higher energy feature for Cl^−^ anions at about 1:0.7. The unique behavior of the TPB anion is correlated with the unique electrostatic surface potential which shows a positive surface surrounding this anion. Presumably the positive surface charge of the TPB anion and the positive charge of the dye cation disrupt aggregation in the solid state due to electrostatic repulsion separating the π-conjugated systems. Attempts to obtain emission spectra from the solid-state films proved to be difficult due to a weak emission signal.

## 3. Materials and Methods

All commercially obtained reagents and solvents were used as received without further purification. Thin-layer chromatography (TLC) was conducted with Sorbtech silica XHL TLC glass backed plates with a UV indication. TLCs were visualized with UV light (254 nm). Silica gel column chromatography was performed using silica gel from Sorbent Tech P60, and pre-packed Normal Phase Disposable RediSep columns (Teledyne) on a Combi-Flash Rf+ system. Neutral alumina chromatography was carried out with pre-packed disposable RediSep columns (Teledyne) with 40–60 µm particle size with a 230–400 mesh. ^1^H and ^19^F NMR spectra were recorded on a Bruker Avance-300 (300 MHz) spectrometer and a Bruker Avance-500 (500 MHz) spectrometer and are reported in ppm using solvent as an internal standard (*d*_6_-DMSO at 2.5 ppm). Data are reported as: s = singlet, d = doublet, t = triplet, q = quartet, p = pentet, m = multiplet, b = broad, ap = apparent, dd = doublet of doublets; coupling constant(s) in Hz; integration. UV-Vis Spectra were measured with a Cary 5000 UV-Vis-NIR spectrometer. HRMS spectra were obtained with a QTOF HRMS utilizing nanospray ionization. The mass analyzer was set to the 200-2000 Da range. Infrared spectra were recorded with an Agilent Cary 660 ATR-FTIR. Thin films of each dye were prepared with a spin coater (Laurell Technologies Corporation, Model WS-650MZ-23NPPB) on VWR 18 × 18 mm micro cover glass slides. Acquisition of emissive data was obtained using a Horiba LabRam Spectrometer with 785 nm diode laser excitation and dye concentrations of 1 × 10^-5^ M. The quantum efficiency of the detector was accounted for when measuring emission profiles. The relative quantum yields were obtained using the following equation:

(1) $$\Phi_{Sample}=\Phi_{Standard}*\frac{E_{Sample}}{E_{Standard}}*\frac{A_{Standard}}{A_{Sample}}*\frac{\eta_{Sample}^{2}}{\eta_{Standard}^{2}}\;$$

For the equation above, E is the sum of emission intensities and A is maximum absorbance. The refractive index of the solvent used is accounted for with η, and Φ denotes the quantum yield [42]. The standard used to obtain the relative quantum yields was indocyanine green (ICG) taking the quantum yield to be 14% in H_2_O as previously reported [43].

(*Z*)-1-methyl-3-((2*E*,4*E*)-5-(1-methyl-2-phenylindolizin-3-yl)penta-2,4-dien-1-ylidene)-2-phenyl-3H-indolizin-4-ium chloride (C5-Cl): To a round bottom flask equipped with a stir bar, C5-ClO_4_ (1.00 g, 1.73 mmol) was added and dissolved in fluorobenzene (0.35 M, 5.0 mL). Tetrabutylammonium chloride (0.47 g, 1.73 mmol) was then added. The mixture was allowed to stir at room temperature overnight. The reaction mixture was then filtered through syringe filters (MicroSolv Nylon, 13 mm diameter, 0.45 μm) to separate the fluorobenzene soluble material from the fine precipitate. The precipitate was then washed from the syringe filters with DCM. The DCM soluble material was then subjected to a silica gel column with a gradient of 100% DCM to 50:50 DCM:methanol, then 50:50 MeCN:H_2_O to fully elute the compound. After concentration, the product was filtered through celite with DCM to remove silica that was dissolved by methanol on the column. The product was isolated as a red solid (0.84 g, 95%). ^1^H NMR (300 MHz, DMSO) δ 9.18 (d, *J* = 6.9 Hz, 2H), 8.01 (d, *J* = 13.6 Hz, 2H), 7.91 (d, *J* = 8.6 Hz, 2H), 7.70–7.54 (m, 8H), 7.37–7.26 (m, 8H), 5.91 (t, *J* = 12.5 Hz, 1H), 2.14 (s, 6H). ^13^C NMR was not obtained due to sparing solubility. IR (neat, cm^-1^): 2800 (br), 2330, 1750 (br), 1619, 1517. HRMS (ESI) *m/z* calculated for C_35_H_29_N_2_ [M]^+^: 477.2331, found 477.2315.

(*Z*)-1-methyl-3-((2*E*,4*E*)-5-(1-methyl-2-phenylindolizin-3-yl)penta-2,4-dien-1-ylidene)-2-phenyl-3*H*-indolizin-4-ium nitrate (C5-NO_3_): To a round bottom flask equipped with a stir bar, C5-Cl (0.020 g, 0.04 mmol) was added and dissolved in acetonitrile (0.008 M, 5.0 mL). Silver nitrate (0.007 g, 0.04 mmol) was then added to the mixture. The reaction mixture was then allowed to stir at room temperature for 5 minutes. The reaction mixture was concentrated and directly subjected to neutral alumina column chromatography with a gradient from 100% DCM to 80:20 dichloromethane:methanol. The concentrated product was filtered through celite with DCM to remove alumina that was dissolved by methanol on the column. The product was isolated as a red solid (0.013 g, 60%). ^1^H NMR (300 MHz, DMSO) δ 9.16 (d, *J* = 6.9 Hz, 2H), 7.99 (d, *J* = 13.6 Hz, 2H), 7.89 (d, *J* = 8.6 Hz, 2H), 7.68–7.54 (m, 8H), 7.35–7.31 (m, 8H), 5.90 (t, *J* = 12.5 Hz, 1H), 2.13 (s, 6H). ^13^C NMR was not obtained due to sparing solubility. IR (neat, cm^-1^): 2700 (br), 2330, 2123, 1707 (br), 1619, 1559, 1517. HRMS (ESI) *m/z* calculated for C_35_H_29_N_2_ [M]^+^: 477.2331, found 477.2357.

(Z)-1-methyl-3-((2E,4E)-5-(1-methyl-2-phenylindolizin-3-yl)penta-2,4-dien-1-ylidene)-2-phenyl-3H-indolizin-4-ium hexafluorophosphate (C5-PF_6_): To a round bottom flask equipped with a stir bar, C5-Cl (0.030 g, 0.06 mmol) was added and dissolved in acetonitrile (0.01 M, 5.0 mL). Silver hexafluorophosphate (0.012 g, 0.06 mmol) was then added to the mixture. The reaction mixture was allowed to stir at room temperature for 5 minutes. The reaction mixture was concentrated then subjected to neutral alumina column chromatography with a gradient of 100% DCM to 80:20 dichloromethane:methanol. The concentrated product was filtered through celite with DCM to remove any alumina that was dissolved by methanol on the column. The product was isolated as a red solid (0.031 g, 79%). ^1^H NMR (300 MHz, DMSO) δ 9.09 (d, J = 6.9 Hz, 2H), 8.00 (d, J = 13.6 Hz, 2H), 7.88 (d, J = 8.6 Hz, 2H), 7.69–7.54 (m, 8H), 7.33–7.26 (m, 8H), 5.84 (t, J = 12.5 Hz, 1H), 2.15 (s, 6H). ^19^F NMR (471 MHz, DMSO) δ -70.16 (d, J = 711.2 Hz). ^13^C NMR was not obtained due to sparing solubility. IR (neat, cm^-1^): 3100 (br), 2800 (br), 1800 (br), 1653, 1540. HRMS (ESI) m/z calculated for C_35_H_29_N_2_ [M]^+^: 477.2331, found 477.2315.

(Z)-1-methyl-3-((2E,4E)-5-(1-methyl-2-phenylindolizin-3-yl)penta-2,4-dien-1-ylidene)-2-phenyl-3H-indolizin-4-ium bis((trifluoromethyl)sulfonyl)amide (C5-TFSI): To a round bottom flask equipped with a stir bar, C5-Cl (0.03 g, 0.06 mmol) was added and dissolved in acetonitrile (0.012 M, 5.0 mL). Silver bis((trifluoromethyl)sulfonyl)amide (0.023 g, 0.06 mmol) was then added to the mixture. The reaction mixture was then allowed to stir at room temperature for 5 minutes. The reaction mixture was concentrated and subjected to silica gel column chromatography with a gradient of 100% DCM to 50:50 dichloromethane:methanol. The product was concentrated and filtered through celite with DCM to remove silica that was dissolved by methanol on the column. The product was isolated as a red solid (0.04 g, 87%). ^1^H NMR (300 MHz, DMSO) δ 9.16 (d, J = 6.9 Hz, 2H), 8.00 (d, J = 13.6 Hz, 2H), 7.88 (d, J = 8.6 Hz, 2H), 7.68–7.56 (m, 8H), 7.37–7.25 (m, 8H), 5.73 (t, J = 12.5 Hz, 1H), 2.15 (s, 6H). ^19^F NMR (471 MHz, DMSO) δ -78.70. ^13^C NMR was not obtained due to sparing solubility. IR (neat, cm^-1^): 2800 (br), 2335, 1750 (br), 1618, 1518. HRMS (ESI) m/z calculated for C_35_H_29_N_2_ [M]^+^: 477.2331, found 477.2315.

(Z)-1-methyl-3-((2E,4E)-5-(1-methyl-2-phenylindolizin-3-yl)penta-2,4-dien-1-ylidene)-2-phenyl-3H-indolizin-4-ium tetraphenylborate (C5-TPB): To a round bottom flask equipped with a stir bar, C5-Cl (0.02 g, 0.04 mmol) was added and dissolved in acetonitrile (0.008 M, 5.0 mL). Sodium tetraphenylborate (0.013 g, 0.04 mmol) was then added to the mixture. The reaction mixture was allowed to stir at room temperature for 5 minutes. The reaction mixture was concentrated and subjected to neutral alumina column chromatography with a gradient of 100% DCM to 80:20 dichloromethane:methanol. The concentrated product was filtered through celite with DCM to remove any alumina that was dissolved by methanol on the column. The product was isolated as a red solid (0.026 g, 84%). ^1^H NMR (300 MHz, DMSO) δ 9.17 (d, J = 6.9 Hz, 2H), 8.01 (d, J = 13.6 Hz, 2H), 7.90 (d, J = 8.6 Hz, 2H), 7.70–7.54 (m, 8H), 7.37–7.18 (m, 10H), 6.95–6.82 (m, 12H), 6.80–6.77 (m, 6H), 5.93 (t, J = 12.5 Hz, 1H), 2.14 (s, 6H). ^13^C NMR was not obtained due to sparing solubility. IR (neat, cm^-1^): 2900 (br), 2336, 2152, 1857 (br), 1618, 1518. HRMS (ESI) m/z calculated for C_35_H_29_N_2_ [M]^+^: 477.2331, found 477.2315.

(*Z*)-1-methyl-3-((2*E*,4*E*)-5-(1-methyl-2-phenylindolizin-3-yl)penta-2,4-dien-1-ylidene)-2-phenyl-3*H*-indolizin-4-ium tetra(3,5-bis(trifluoromethyl)phenyl)borane (C5-BARF): To a round bottom flask equipped with a stir bar, C5-Cl (0.02 g, 0.04 mmol) was added and dissolved in acetonitrile (0.008 M, 5.0 mL). Sodium tetra(3,5-bis(trifluoromethyl)phenyl)borane (0.04 g, 0.04 mmol) was then added to the mixture. The reaction mixture was allowed to stir at room temperature for 5 minutes. The reaction mixture was concentrated and directly subjected to neutral alumina column chromatography with a gradient of 100% DCM to 80:20 dichloromethane:methanol. After concentration, the product was filtered through celite with DCM to remove alumina that was dissolved by methanol on the column. The product was isolated as a red solid (0.03 g, 84%). ^1^H NMR (300 MHz, DMSO) δ 9.17 (d, *J* = 6.9 Hz, 2H), 8.01 (d, *J* = 13.6 Hz, 2H), 7.90 (d, *J* = 8.6 Hz, 2H), 7.74–7.54 (m, 18H), 7.37–7.26 (m, 6H), 5.93 (t, *J* = 12.5 Hz, 1H), 2.14 (s, 6H). ^19^F NMR (471 MHz, DMSO) δ -61.60.^13^C NMR was not obtained due to sparing solubility. IR (neat, cm^-1^): 2918, 2852, 2800 (br), 2330, 2121, 1771, 1619, 1559, 1520. HRMS (ESI) *m/z* calculated for C_35_H_29_N_2_ [M]^+^: 477.2331, found 477.2303.

## 4. Conclusions

We have synthesized a series of seven compounds that differ only in anion properties. Anion selection has a dramatic effect on molar absorptivity behaviors depending on solvent selection, Stokes shifts variance, altered quantum yields, and solid-state photophysics. These results highlight the critical importance of strictly controlling the anion and the environment around the dye system when comparing optical properties of various dyes in the literature. DCM measurements are found to track with anion size showing that molar absorptivities can be significantly enhanced by a simple non-coordinating anion swap.

## Figures and Tables

**Table 1:** Calculated anion volumes.

Anion	Volume/Mole (cm^3^/mol)	Volume (cm^3^)
**Cl^−^**	36.741	6.1011 x 10^-23^
**NO_3_^−^**	35.092	5.8273 x 10^−23^
**ClO_4_^−^**	52.148	8.6596 x 10^−23^
**PF_6_^−^**	90.531	1.5033 x 10^−22^
**TFSI**	130.771	2.1716 x 10^−22^
**TPB**	246.294	4.0899 x 10^−22^
**BARF**	414.785	6.8878 x 10^-22^

**Table 2:** Optical properties of each dye in acetonitrile and dichloromethane.

Dye	Solvent	Molar Abs. (M^−1^cm^−1^)	λ_max_^abs^ (nm)	λ_max_^emis^ (nm)	Φ (%)	MB (ε × Φ)	Stokes Shift (nm | eV | cm^−1^)
**C5-ClO_4_**	MeCN	140,000	810	848	1.2	1,700	38 | 0.07 | 553
	DCM	188,000	825	868	2.2	4,100	43 | 0.07 | 600
**C5-Cl**	MeCN	87,000	810	866	<1	<870	56 | 0.10 | 798
	DCM	170,000	821	849	<1	<1,700	28 | 0.05 | 402
**C5-NO_3_**	MeCN	66,000	810	842	<1	<660	32 | 0.06 | 469
	DCM	120,000	825	856	<1	<1,200	31 | 0.06 | 439
**C5-PF_6_**	MeCN	106,000	810	836	<1	<1,060	26 | 0.05 | 384
	DCM	186,000	826	862	<1	<1,860	36 | 0.07 | 506
**C5-TFSI**	MeCN	141,000	810	838	1.1	1,551	28 | 0.06 | 413
	DCM	207,000	826	866	<1	<2,070	40 | 0.07 | 559
**C5-TPB**	MeCN	106,000	810	840	1.2	1,272	30 | 0.06 | 441
	DCM	148,000	826	867	<1	<1,480	41 | 0.07 | 573
**C5-BARF**	MeCN	138,000	810	836	<1	<1,380	26 | 0.05 | 384
	DCM	238,000	826	861	<1	<2,380	35 | 0.06 | 492

**Table 3:** Optical properties of each dye on thin film.

Dye	λ_max_^abs^ (nm)	Abs. High E: Low E Feature Ratio
**C5-ClO_4_**	875	1:1
**C5-Cl**	750^a^	1:0.7
**C5-NO_3_**	735^a^	1:0.8
**C5-PF_6_**	778^a^	1:0.9
**C5-TFSI**	722^a^	1:0.7
**C5-TPB**	878^b^	0.8:1
**C5-BARF**	800^a^	1:1

^a^ Indicates a lower energy shoulder is present near 875 nm. ^b^ Indicates a higher energy shoulder is present near 805 nm.

## Supplementary Materials

The following are available online at http://www.mdpi.com/1420-3049/23/12/3051/s1, Table S1: Mulliken charges on NO_3_^-^ atoms. Table S2: Mulliken charges on ClO_4_^-^ atoms. Table S3: Mulliken charges on PF_6_^-^ atoms. Table S4: Mulliken charges on TFSI atoms. Table S5: Mulliken charges on TPB atoms. Table S6: Mulliken charges on BARF atoms. Figure S1: ^1^H NMR in DMSO of C5-Cl. Figure S2: ^1^H NMR in DMSO of C5-NO_3_. Figure S3: ^1^H NMR in DMSO of C5-PF_6_. Figure S4: ^19^F NMR in DMSO of C5-PF_6_. Figure S5: ^1^H NMR in DMSO of C5-TFSI. Figure S6: ^19^F NMR in DMSO of C5-TFSI. Figure S7: ^1^H NMR in DMSO of C5-TPB. Figure S8: ^1^H NMR in DMSO of C5-BARF. Figure S8: ^19^F NMR in DMSO of C5-BARF.
