# Supplementary material for: Counter Anion Effect on the Photophysical Properties of Emissive Indolizine-Cyanine Dyes in Solution and Solid State
Source: Molecules. 2018 Nov 22;23(12):3051. doi: 10.3390/molecules23123051 (PMC6321477; doi:10.3390/molecules23123051)
Supplement: Supplementary file 1 [file molecules-23-03051-s001.pdf]

Counter Anion Effect on the Photophysical Properties of Emissive Indolizine-Cyanine Dyes in Solution and Solid State

Jacqueline N. Gayton,[a,†] Shane Autry,[a,†] Ryan C. Fortenberry,[a] Nathan I. Hammer,[a] and Jared H. Delcamp*,[a]

[a] Department of Chemistry and Biochemistry, University of Mississippi, University, MS, 38677

[†] These authors contributed equally.

* email: delcamp@olemiss.edu

Table of Contents

1. Computational Data
Page 3 Mulliken charges on NO_3^- atoms (Table S1)
Page 3 Mulliken charges on ClO_4^- atoms (Table S2)
Page 3 Mulliken charges on PF_6^- atoms (Table S3)
Page 4 Mulliken charges on TFSI atoms (Table S4)
Page 5 Mulliken charges on TPB atoms (Table S5)
Page 6 Mulliken charges on BARF atoms (Table S6)

2. NMR Characterization Data
Page 7 ^{1}H NMR in DMSO of **C5-Cl** (Figure S1)
Page 8 ^{1}H NMR in DMSO of **C5-NO$_3$** (Figure S2)
Page 9 ^{1}H NMR in DMSO of **C5-PF$_6$** (Figure S3)
Page 10 ^{19}F NMR in DMSO of **C5-PF$_6$** (Figure S4)
Page 11 ^{1}H NMR in DMSO of **C5-TFSI** (Figure S5)
Page 12 ^{19}F NMR in DMSO of **C5-TFSI** (Figure S6)
Page 13 ^{1}H NMR in DMSO of **C5-TPB** (Figure S7)
Page 14 ^{1}H NMR in DMSO of **C5-BARF** (Figure S8)
Page 15 ^{19}F NMR in DMSO of **C5-BARF** (Figure S9)

3. Photophysical Data
Page 16 Full spectrum molar absorptivity and emission plot in MeCN (Figure S10).
Page 16 Full spectrum molar absorptivity and emission plot in MeCN (Figure S11).
Page 17 Concentration versus absorbance plot for **C5-Cl** in DCM (Figure S12).
Page 17 Concentration versus absorbance plot for **C5-NO$_3$** in DCM (Figure S13).
Page 18 Concentration versus absorbance plot for **C5-PF$_6$** in DCM (Figure S14).
Page 18 Concentration versus absorbance plot for **C5-TFSI** in DCM (Figure S15).
Page 19 Concentration versus absorbance plot for **C5-TPB** in DCM (Figure S16).
Page 19 Concentration versus absorbance plot for **C5-BARF** in DCM (Figure S17).
Page 20 Concentration versus absorbance plot for **C5-Cl** in MeCN (Figure S18).
Page 20 Concentration versus absorbance plot for **C5-NO$_3$** in MeCN (Figure S19).
Page 21 Concentration versus absorbance plot for **C5-PF$_6$** in MeCN (Figure S20).
Page 21 Concentration versus absorbance plot for **C5-TFSI** in MeCN (Figure S21).
Page 22 Concentration versus absorbance plot for **C5-TPB** in MeCN (Figure S22).
Page 22 Concentration versus absorbance plot for **C5-BARF** in MeCN (Figure S23).
Page 23 Absorption spectrum of varying concentrations of **C5-Cl** in MeCN (Figure S24).
Page 23 Film absorption spectrum not normalized (Figure S25).
Page 23 Film absorption with **C5-Cl** on glass with varying concentrations of the dye (Figure S26).
Page 24 Film absorption with **C5-TPB** on glass varying dye concentrations (Figure S27).
Page 24 Film absorption with **C5-TPB** and **C5-Cl** on FTO coated glass (Figure S28).

Table S1. Mulliken charges on NO_3^- atoms.

Atom Number	Atom	Charge
1	N	-0.052098
2	O	-0.315967
3	O	-0.315967
4	O	-0.315967

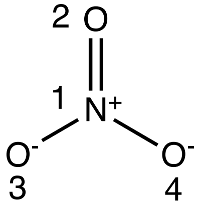

Table S2. Mulliken charges on ClO_4^- atoms.

Atom Number	Atom	Charge
1	Cl	1.351688
2	O	-0.587922
3	O	-0.587922
4	O	-0.587922
5	O	-0.587922

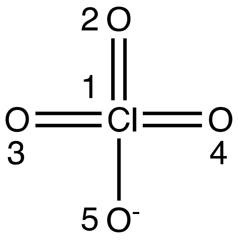

Table S3. Mulliken charges on PF_6^- atoms.

Atom Number	Atom	Charge
1	P	3.403124
2	F	-0.733854
3	F	-0.733854
4	F	-0.733854
5	F	-0.733854
6	F	-0.733854
7	F	-0.733854

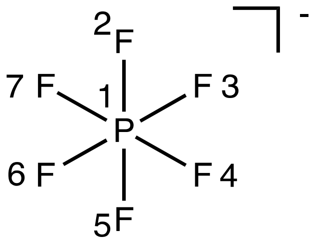

Table S4. Mulliken charges on TFSI atoms.

Atom Number	Atom	Charge
1	S	1.176786
2	C	0.742771
3	F	-0.309487
4	F	-0.294206
5	F	-0.266768
6	N	-0.878432
7	S	1.117281
8	C	0.828531
9	F	-0.315792
10	F	-0.282991
11	F	-0.274752
12	O	-0.556669
13	O	-0.568497
14	O	-0.551996
15	O	-0.565780

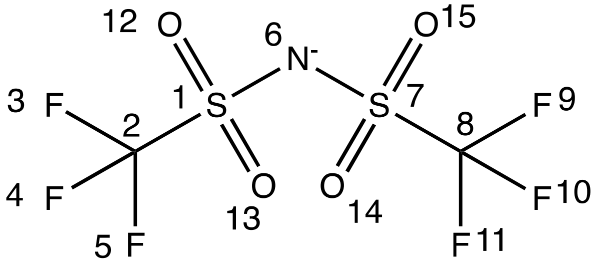

Table S5. Mulliken charges on TPB atoms.

Atom Number	Atom	Charge
1	B	-0.158270
2	C	1.128749
3	C	-0.481455
4	C	-0.418219
5	C	-0.330027
6	C	-0.418219
7	C	-0.481455
8	H	0.178060
9	H	0.145291
10	H	0.143492
11	H	0.145291
12	H	0.178060
13	C	1.128749
14	C	-0.481455
15	C	-0.418219
16	C	-0.330027
17	C	-0.418219
18	C	-0.481455
19	H	0.178060
20	H	0.145291
21	H	0.143492
22	H	0.145291
23	H	0.178060
24	C	1.128749
25	C	-0.481455
26	C	-0.418219
27	C	-0.330027
28	C	-0.418219
29	C	-0.481455
30	H	0.178060
31	H	0.145291
32	H	0.143492
33	H	0.145291
34	H	0.178060
35	C	1.128749
36	C	-0.481455
37	C	-0.418219
38	C	-0.330027
39	C	-0.418219
40	C	-0.481455
41	H	0.178060
42	H	0.145291
43	H	0.143492
44	H	0.145291
45	H	0.178060

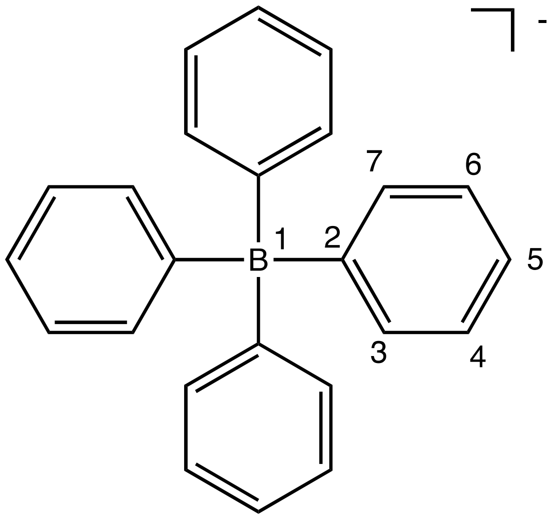

Table S6. Mulliken charges on BARF atoms.

Atom Number	Atom	Charge
1	B	0.246940
2	C	-0.467115
3	C	0.030678
4	C	-1.081474
5	C	-0.353031
6	C	-0.525904
7	C	-0.424496
8	H	0.215602
9	C	1.885359
10	F	-0.322164
11	F	-0.302350
12	F	-0.273043
13	H	0.220926
14	C	1.771129
15	F	-0.296042
16	F	-0.274135
17	F	-0.332978
18	H	0.220021
19	C	-0.337231
20	C	0.010444
21	C	-1.091433
22	C	-0.315577
23	C	-0.594870
24	C	-0.373494
25	H	0.215116
26	C	1.752906
27	F	-0.304481
28	F	-0.268874
29	F	-0.327559
30	H	0.220899
31	C	1.838879
32	F	-0.272759
33	F	-0.301823
34	F	-0.329219
35	H	0.221551
36	C	-0.409728
37	C	-0.022202
38	C	-1.101163
39	C	-0.314471
40	C	-0.573120
41	C	-0.341871
42	H	0.215555
43	C	1.737881
44	F	-0.269514
45	F	-0.304927
46	F	-0.328041
47	H	0.221053
48	C	1.828482
49	F	-0.271783
50	F	-0.300031
51	F	-0.329847
52	H	0.220930
53	C	-0.391629
54	C	-0.316589
55	C	-0.740104
56	C	-0.283551
57	C	-1.233593
58	C	0.015076
59	H	0.219623
60	C	1.870671
61	F	-0.332687
62	F	-0.271987
63	F	-0.297009
64	H	0.220620
65	C	1.887648
66	F	-0.265933
67	F	-0.303000
68	F	-0.330092
69	H	0.214931

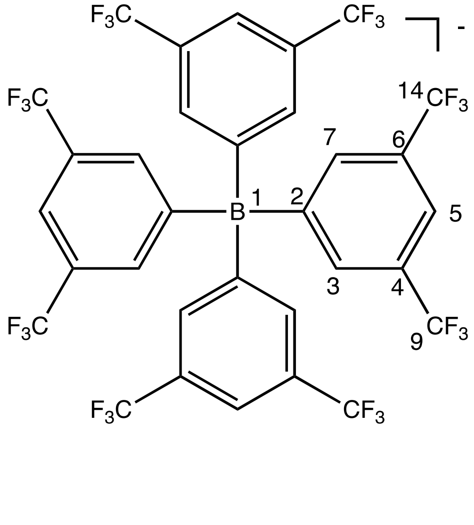

Figure S1. ^{1}H NMR NMR of **C5-BARF** in DMSO.

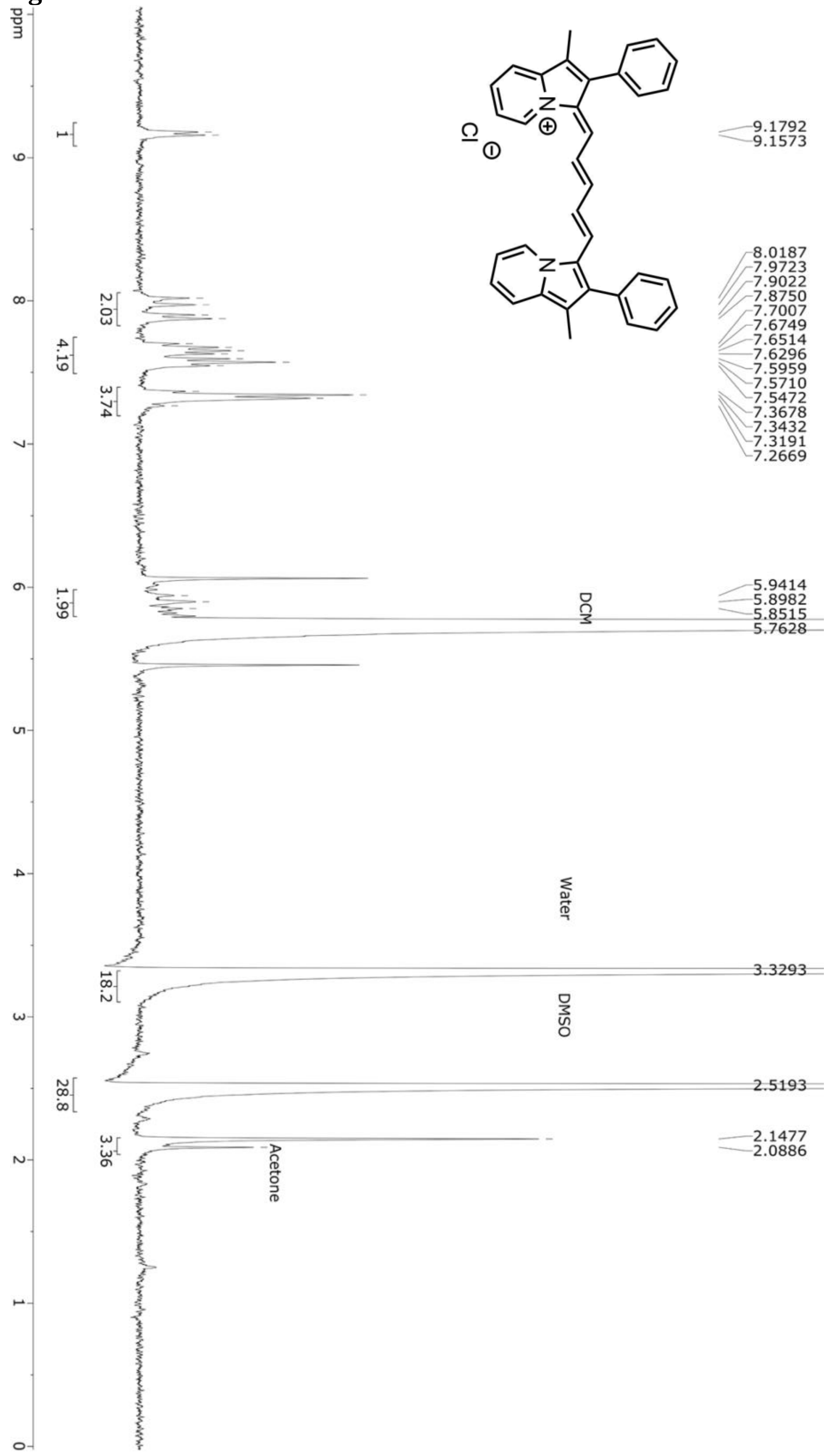

Figure S2. ^{1}H NMR (DMSO) of **C5-NO$_3$**

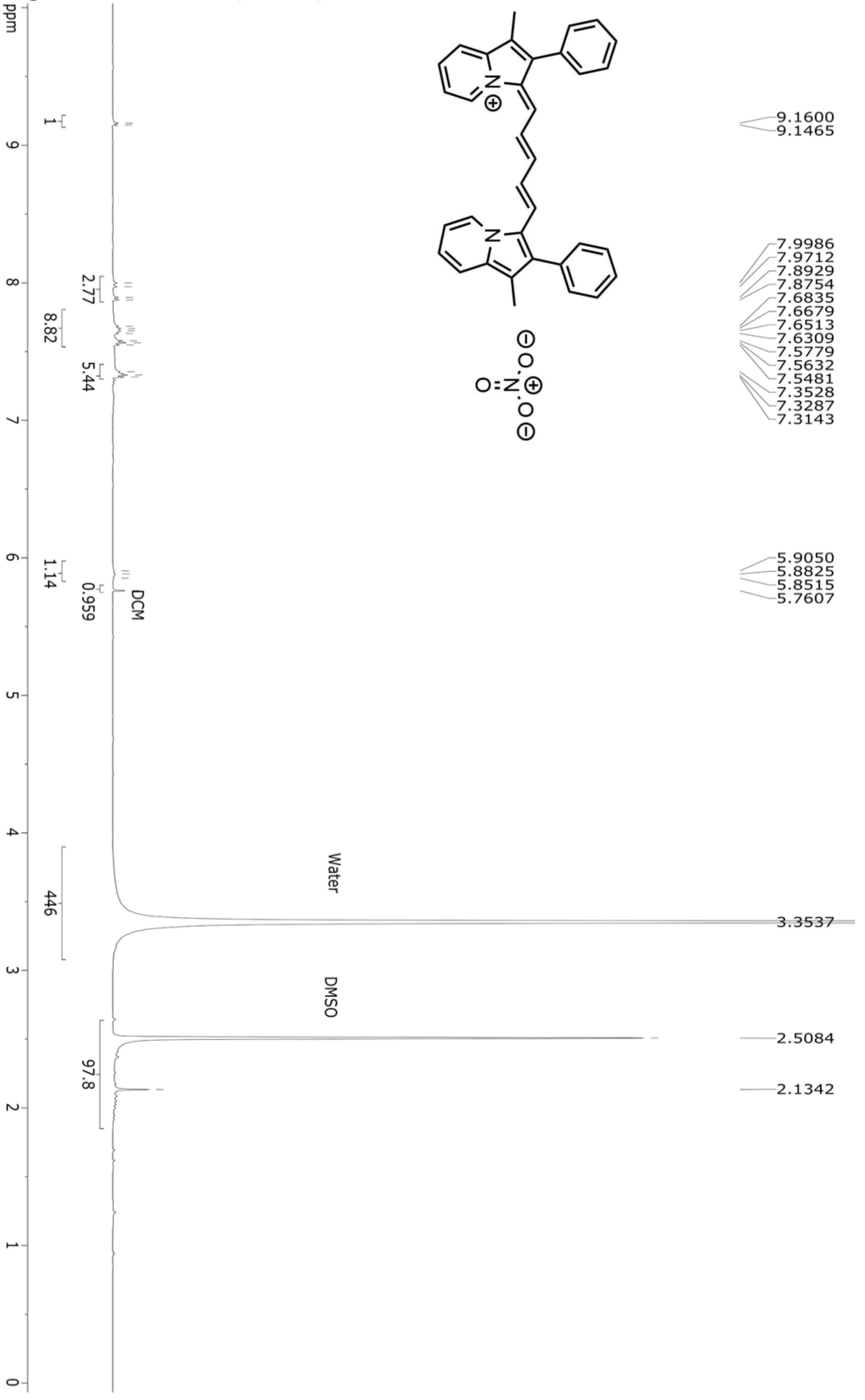

Figure S3. ^{1}H NMR (DMSO) of **C5-PF$_6$**

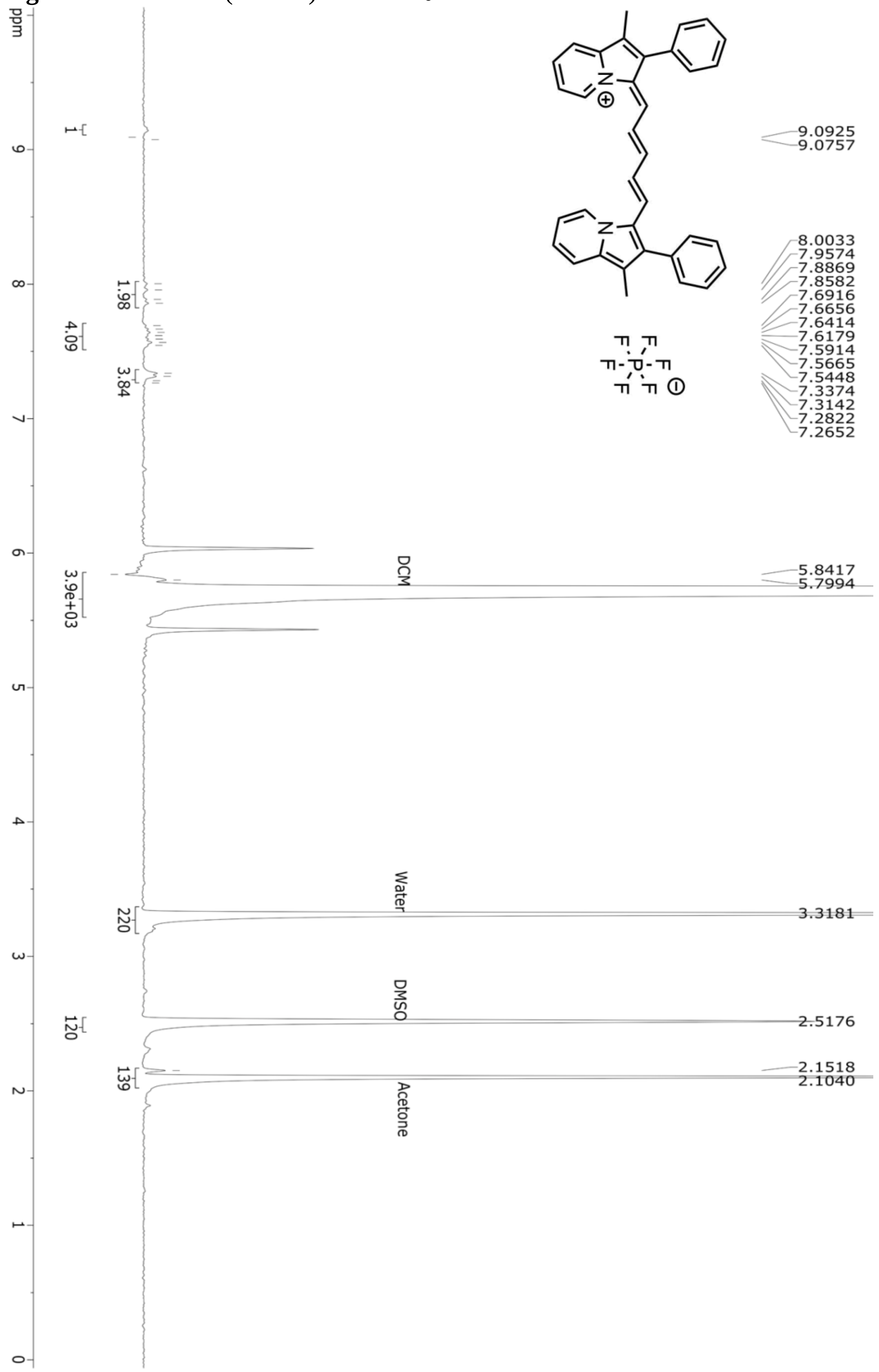

Figure S4. ^{19}F NMR (DMSO) of **C5-PF$_6$**

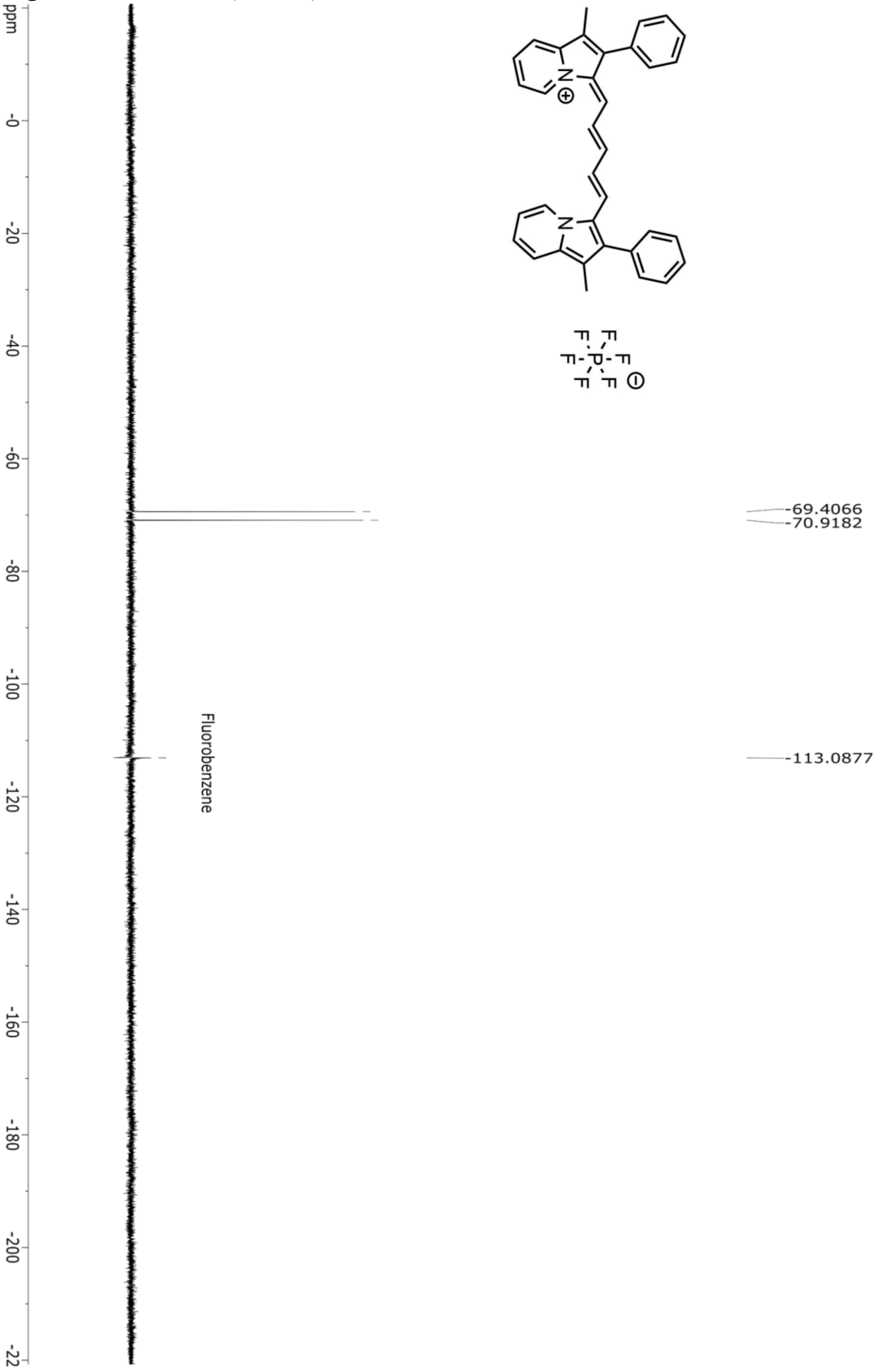

Figure S5. ^{1}H NMR (DMSO) of **C5-TFSI**

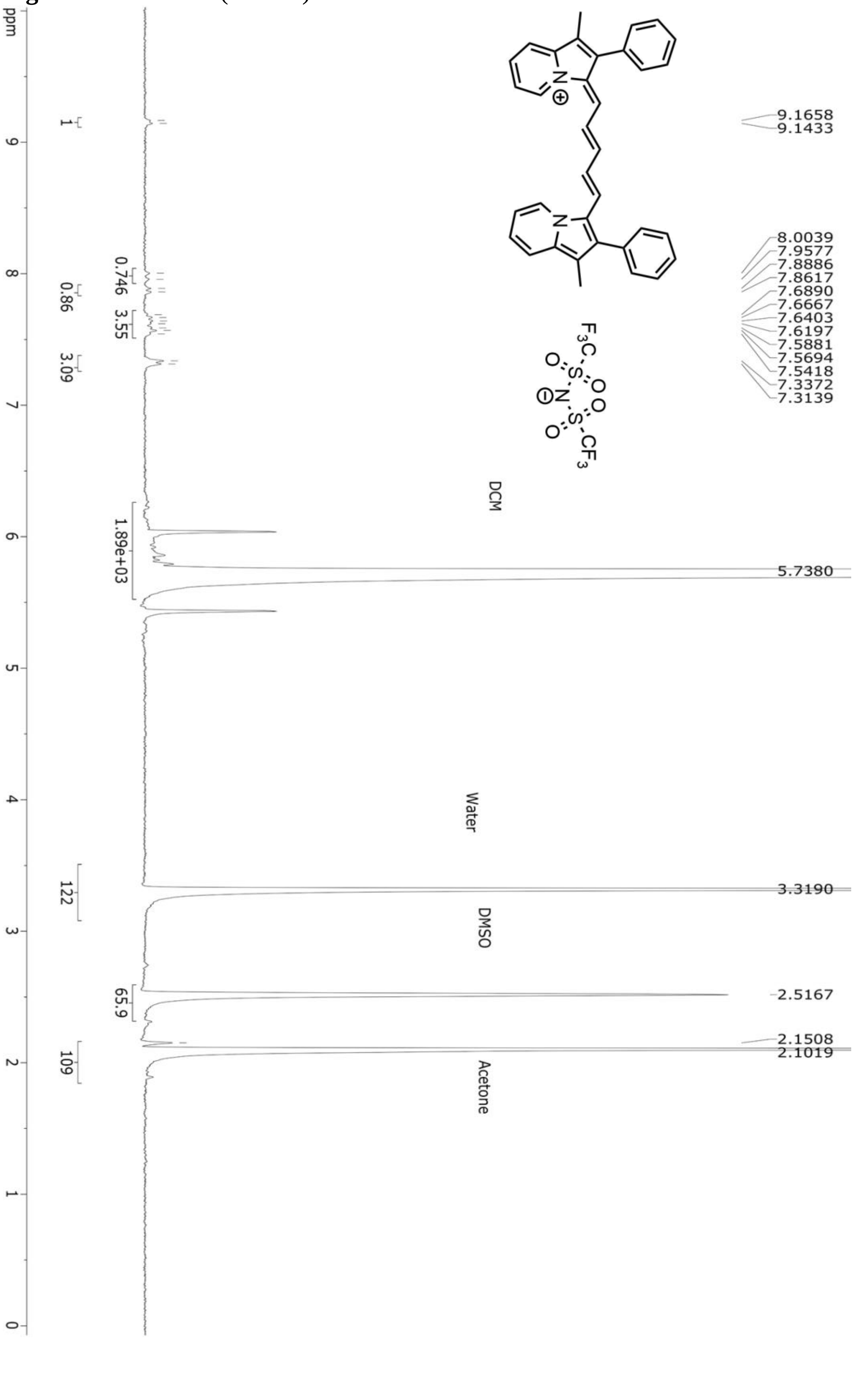

Figure S6. ^{19}F NMR (DMSO) of **C5-TFSI**

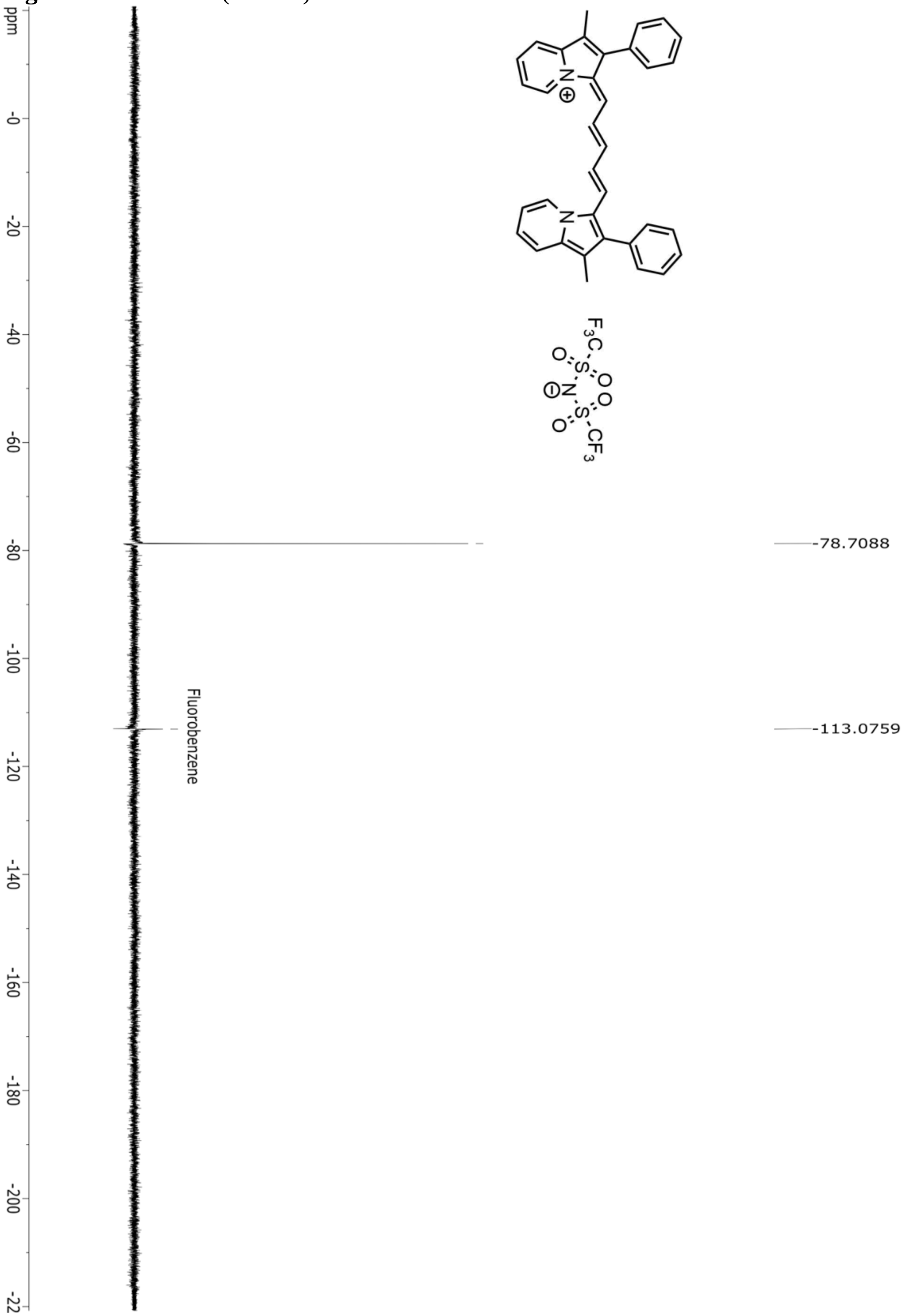

Figure S7. ^1H NMR (DMSO) of **C5-TPB**

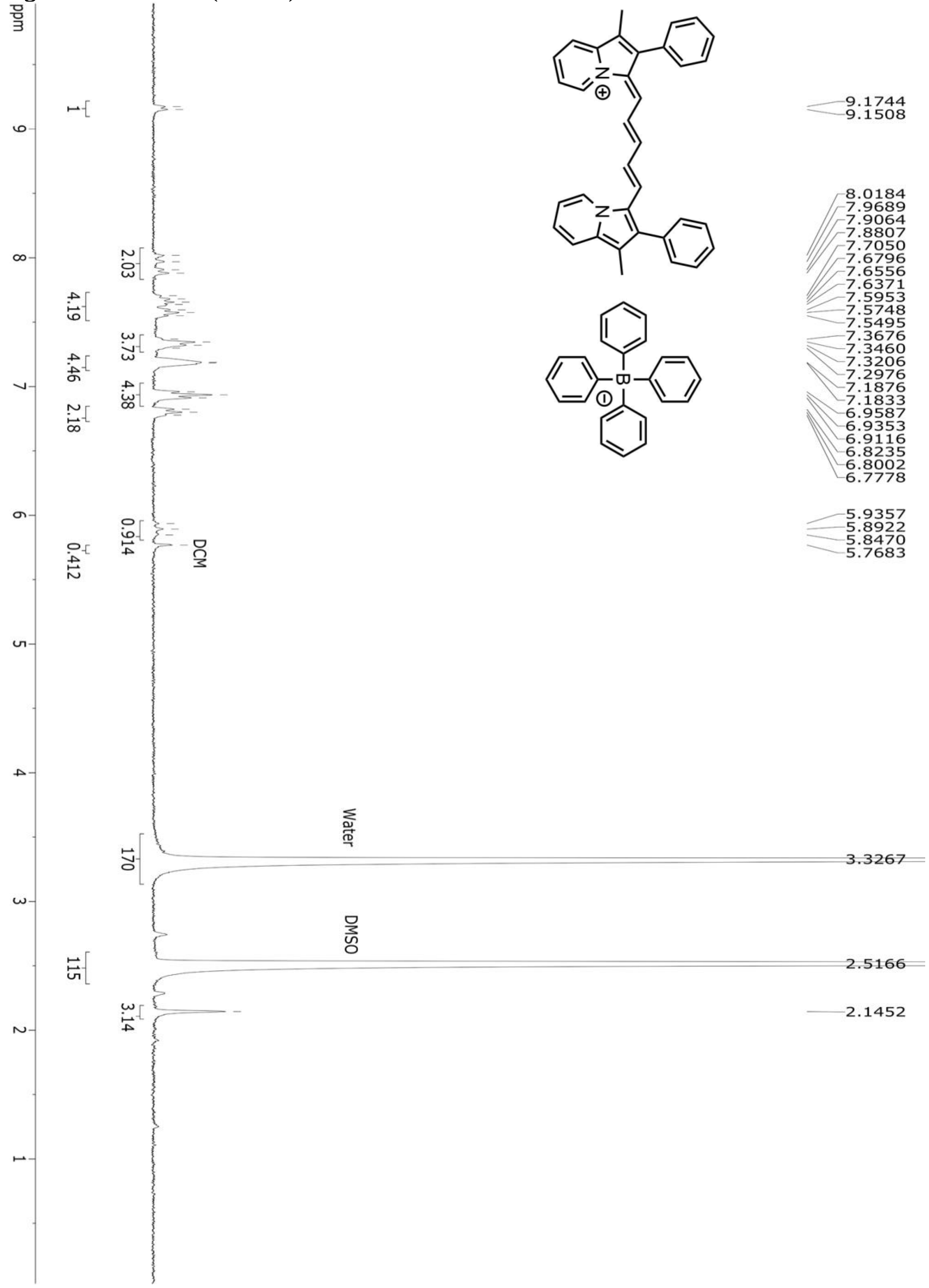

Figure S8. ^{1}H NMR (DMSO) of **C5-BARF**

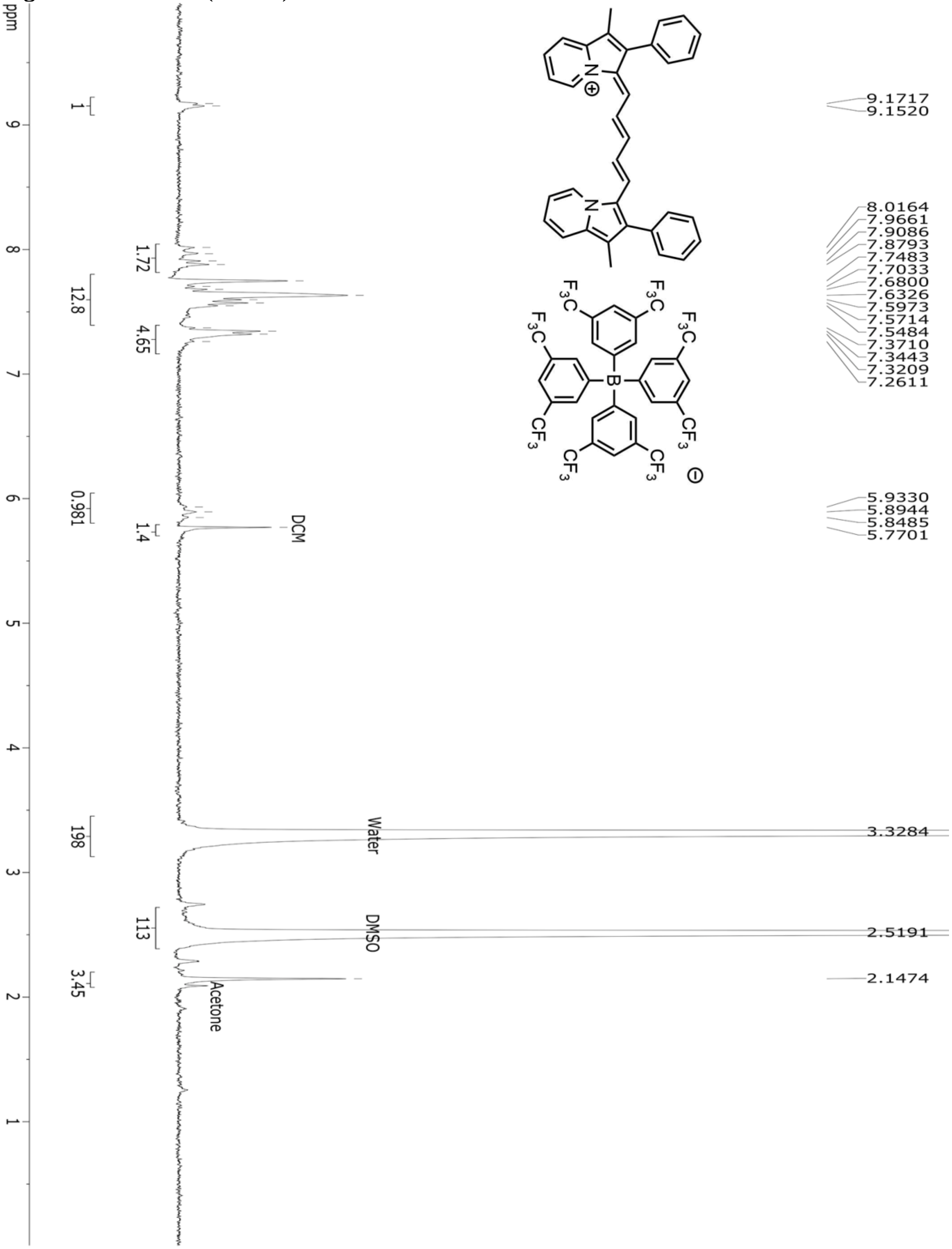

Figure S9. ^{19}F NMR (DMSO) of **C5-BARF**

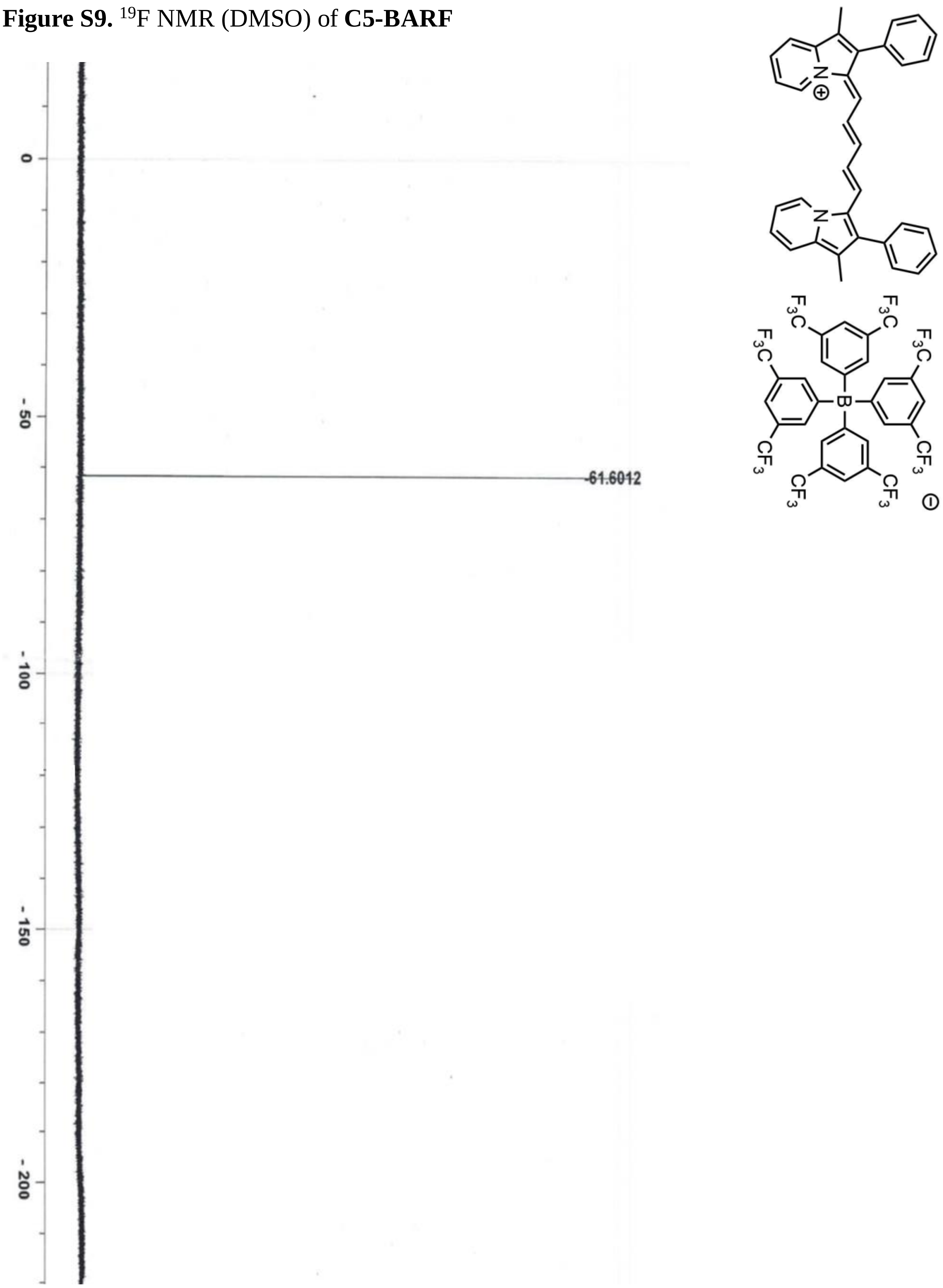

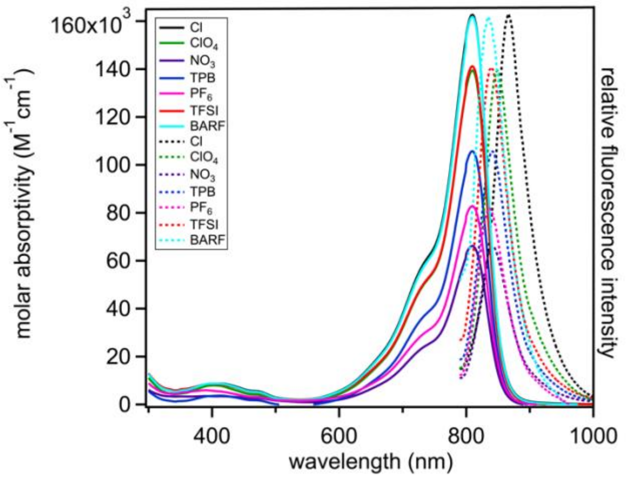

Figure S10. Full spectrum (300-1000 nm) of molar absorptivity and emission plot in MeCN.

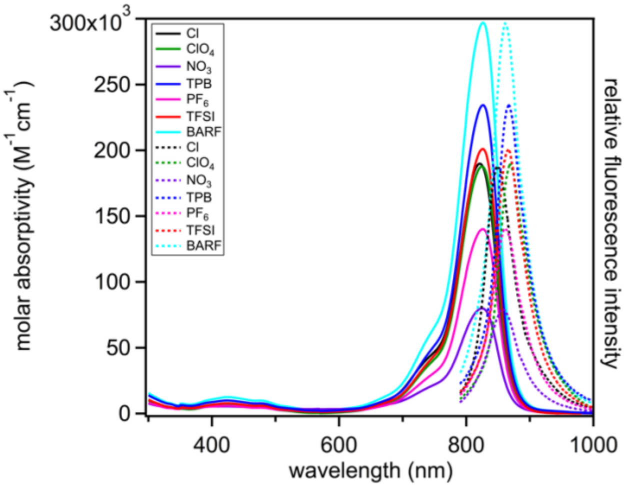

Figure S11. Full spectrum (300-1000 nm) of molar absorptivity and emission plot in DCM.

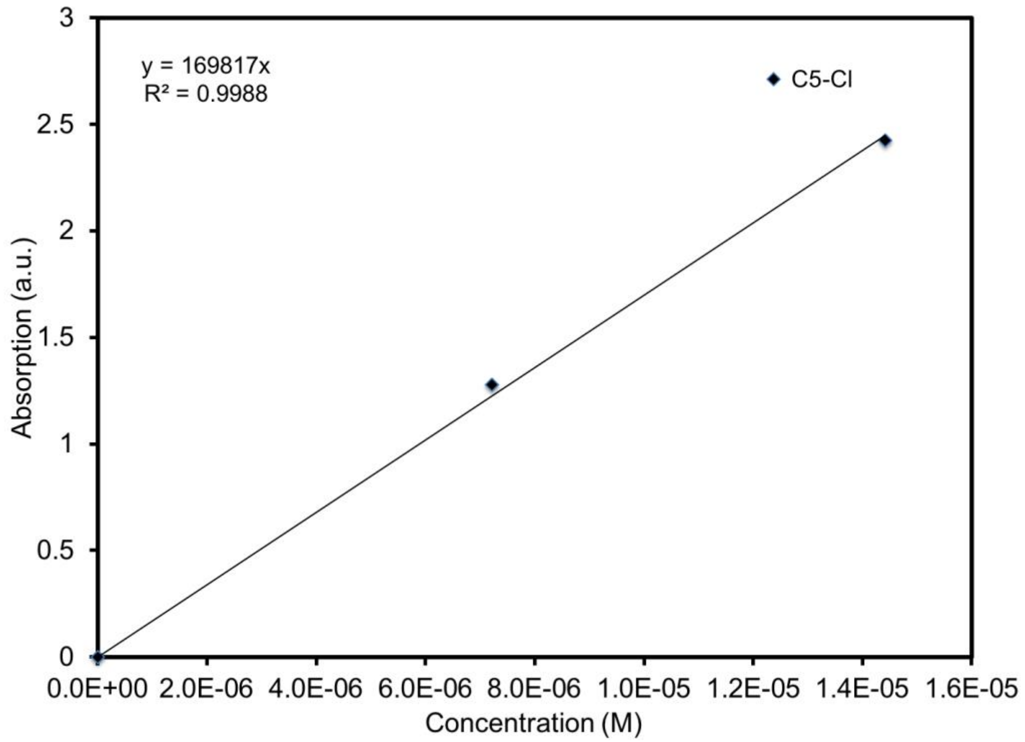

Figure S12. Concentration versus absorbance plot for **C5-Cl** in DCM.

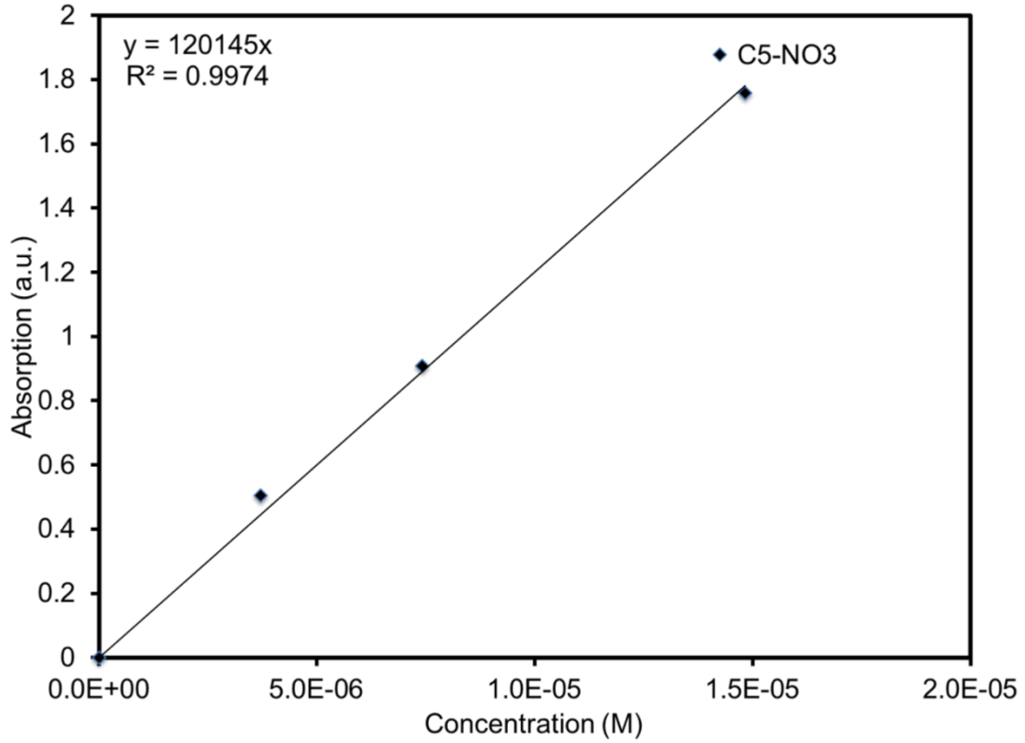

Figure S13. Concentration versus absorbance plot for **C5-NO$_3$** in DCM.

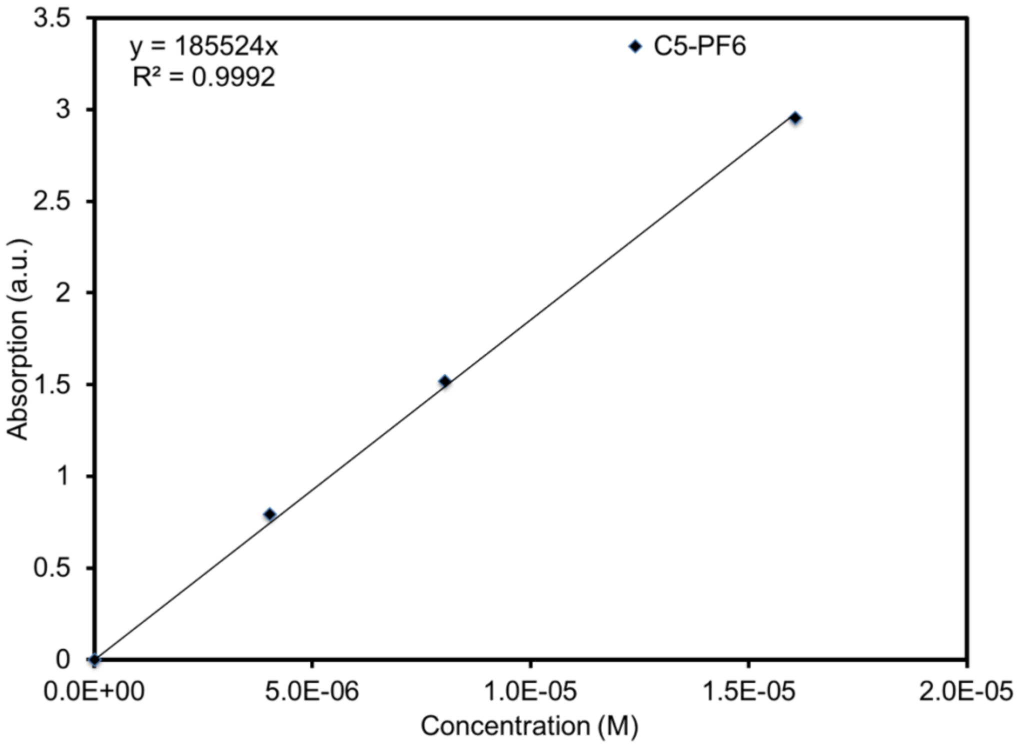

Figure S14. Concentration versus absorbance plot for **C5-PF$_6$** in DCM.

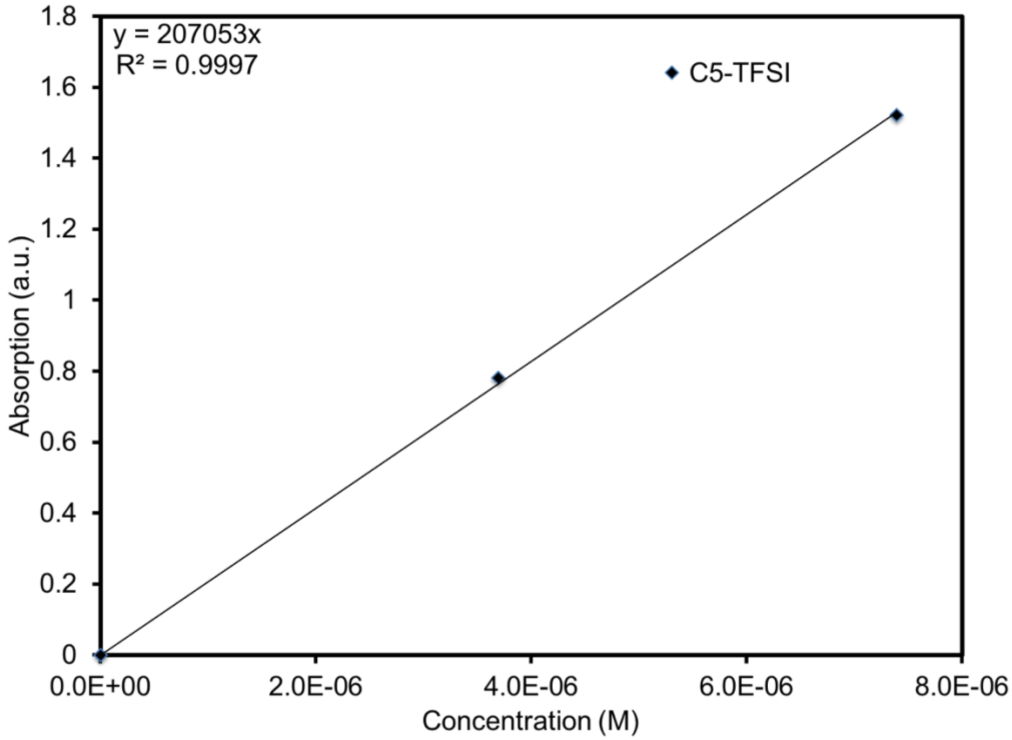

Figure S15. Concentration versus absorbance plot for **C5-TFSI** in DCM.

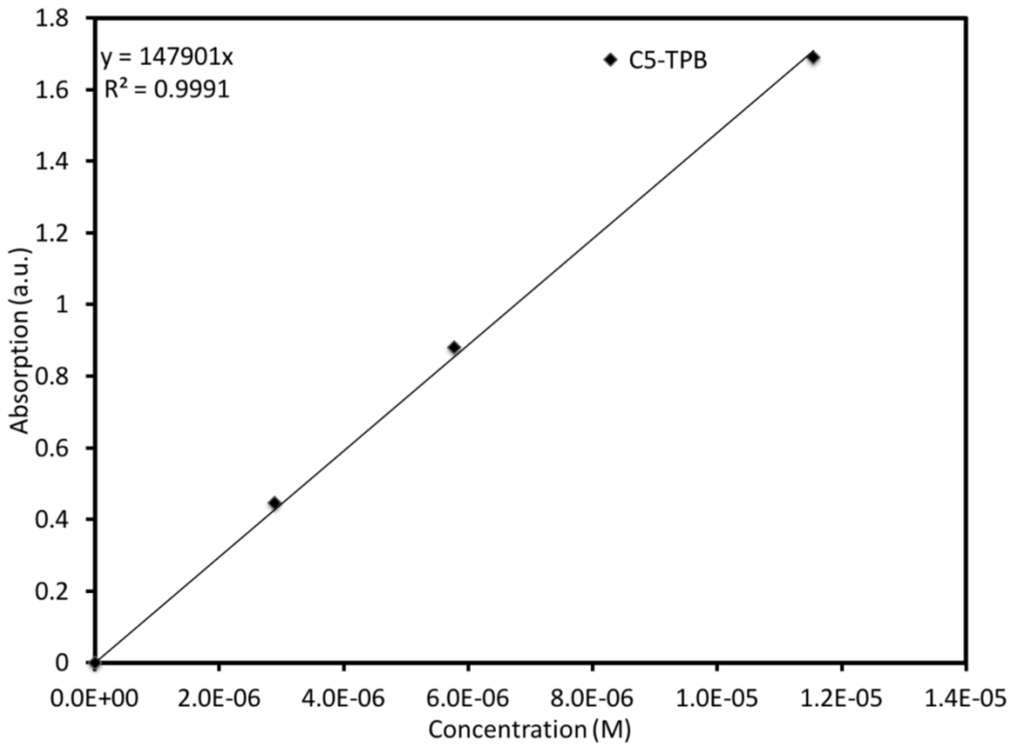

Figure S16. Concentration versus absorbance plot for **C5-TPB** in DCM.

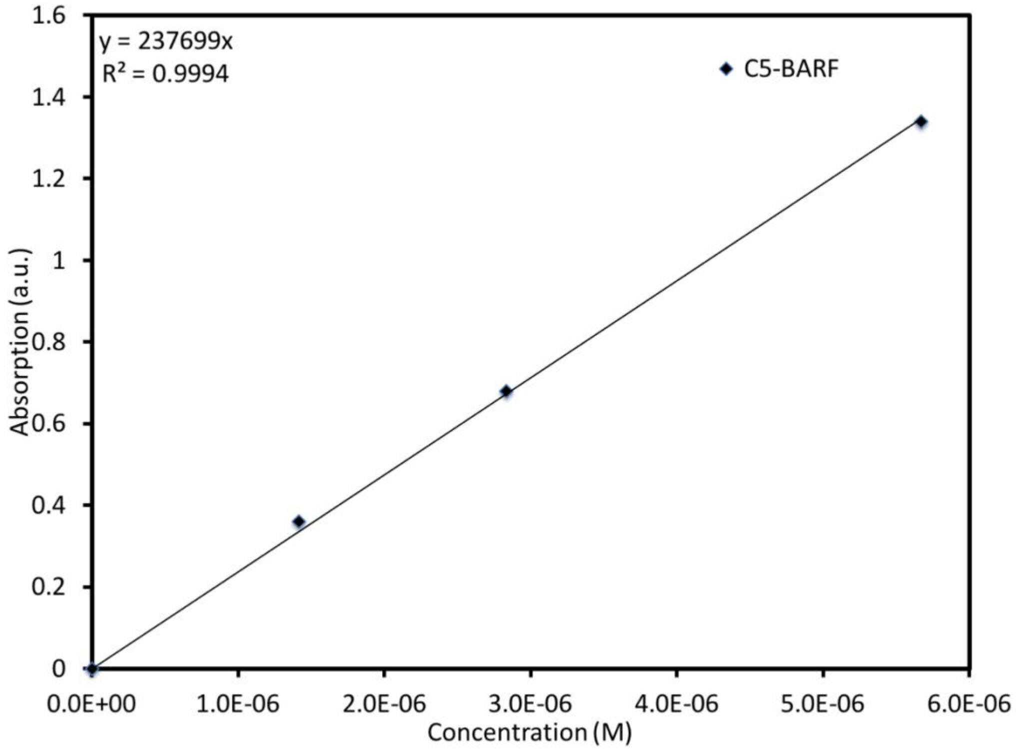

Figure S17. Concentration versus absorbance plot for **C5-BARF** in DCM.

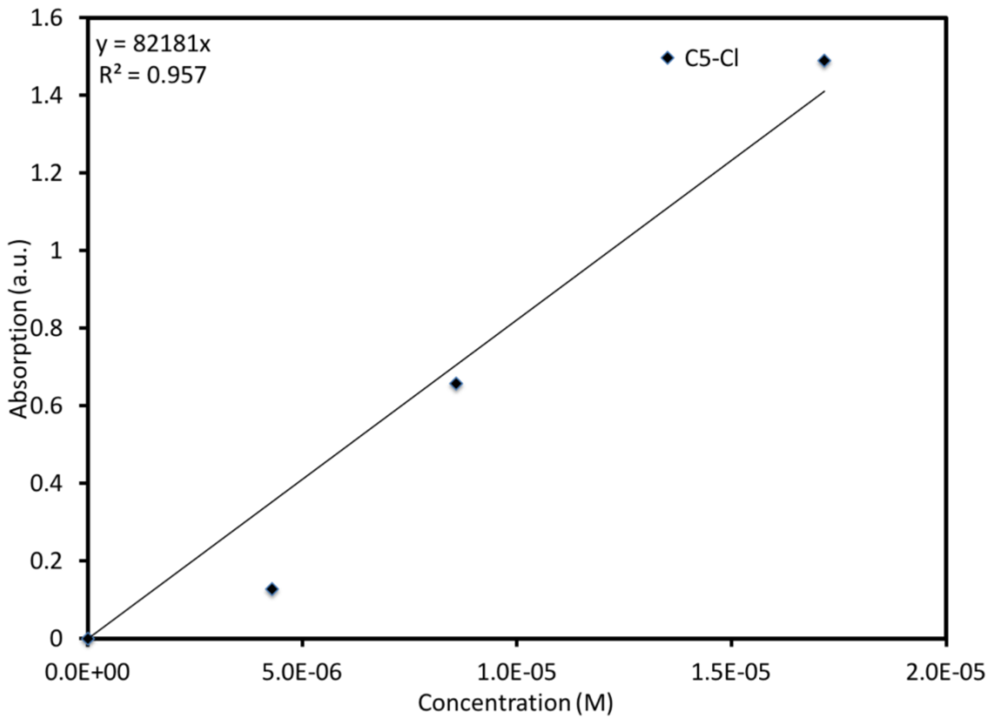

Figure S18. Concentration versus absorbance plot for **C5-Cl** in MeCN. A positive deviation from the Beer-Lambert Law is observed.

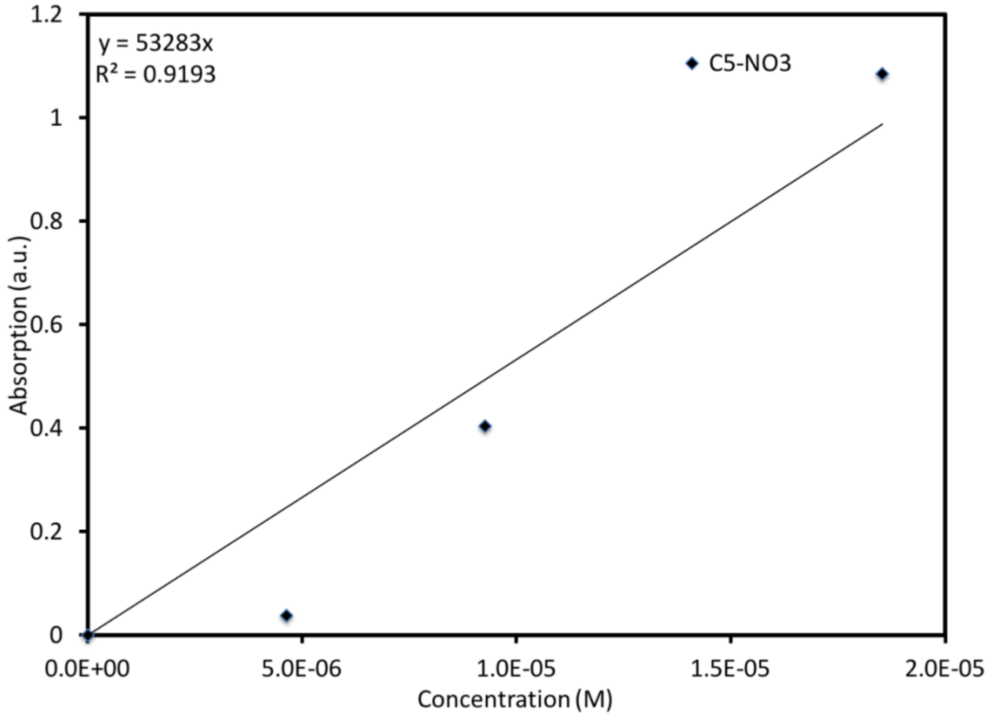

Figure S19. Concentration versus absorbance plot for **C5-NO$_3$** in MeCN. A positive deviation from the Beer-Lambert Law is observed.

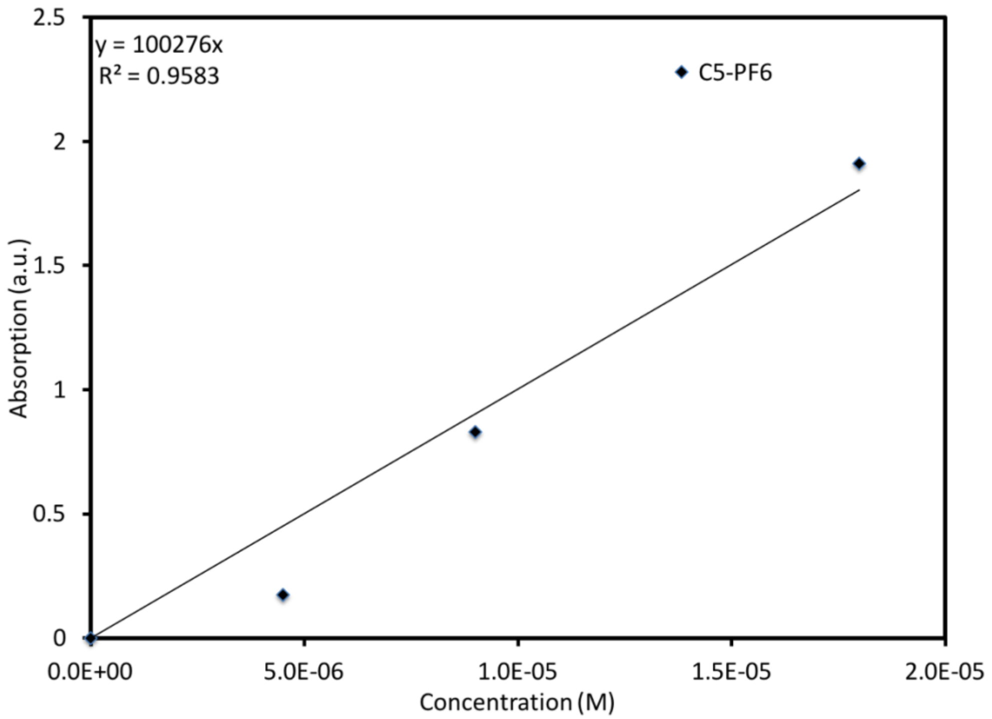

Figure S20. Concentration versus absorbance plot for **C5-PF$_6$** in MeCN. A positive deviation from the Beer-Lambert Law is observed.

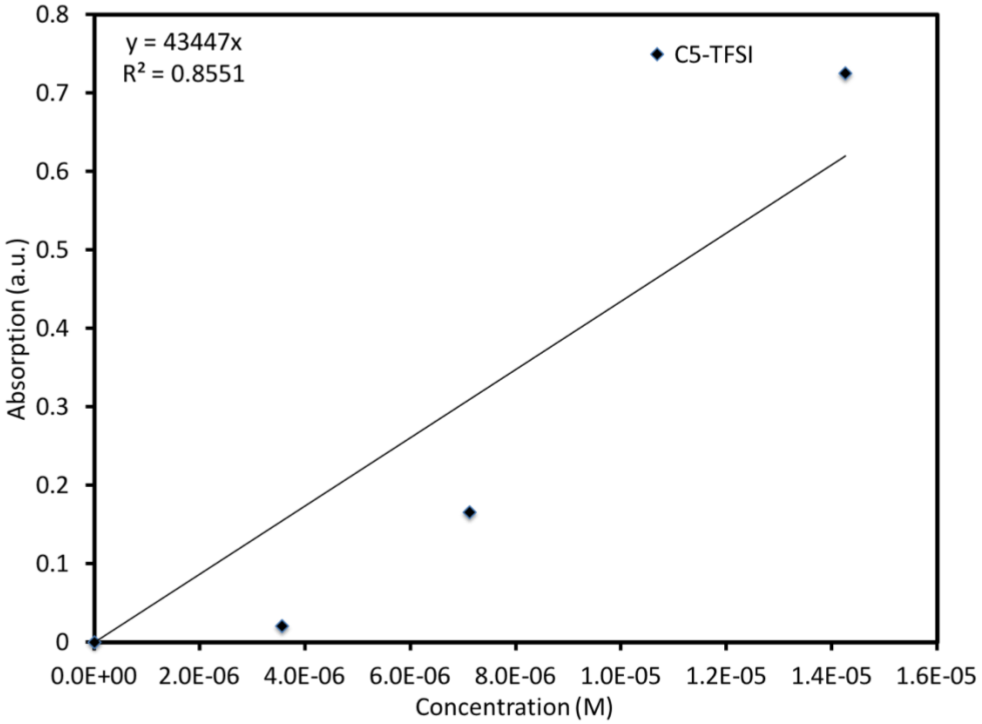

Figure S21. Concentration versus absorbance plot for **C5-TFSI** in MeCN. A positive deviation from the Beer-Lambert Law is observed.

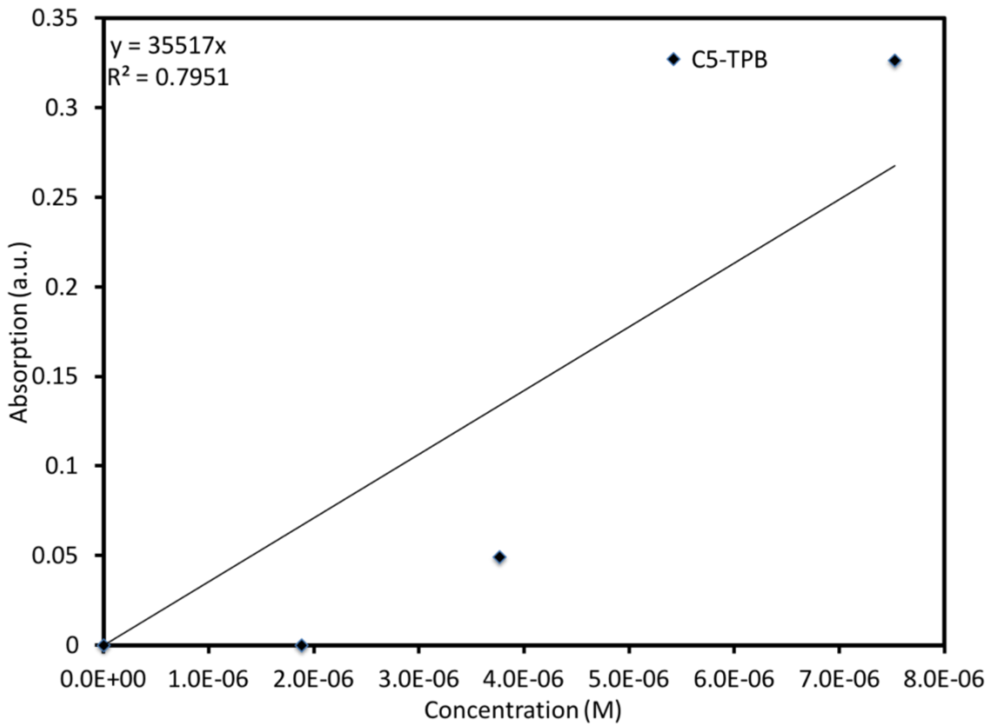

Figure S22. Concentration versus absorbance plot for **C5-TPB** in MeCN. A positive deviation from the Beer-Lambert Law is observed.

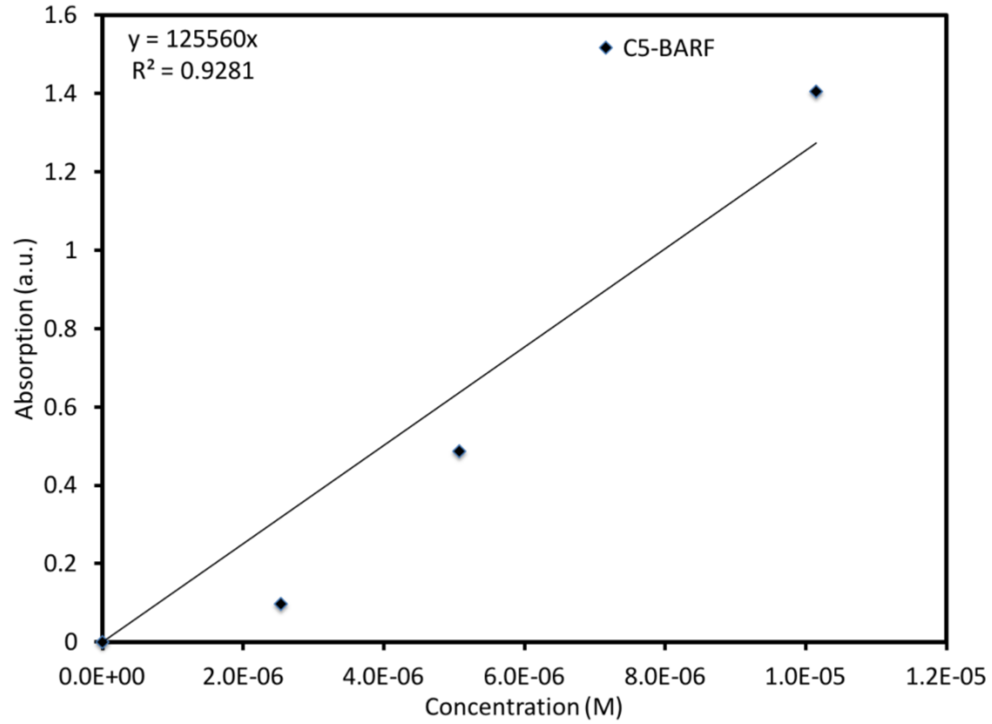

Figure S23. Concentration versus absorbance plot for **C5-BARF** in MeCN. A positive deviation from the Beer-Lambert Law is observed.

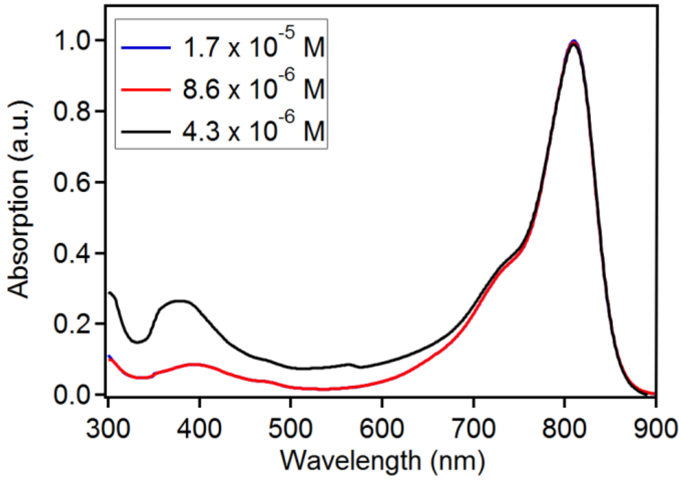

Figure S24. Absorption spectrum of varying concentrations of **C5-Cl** in MeCN showing a change in features as concentration changes.

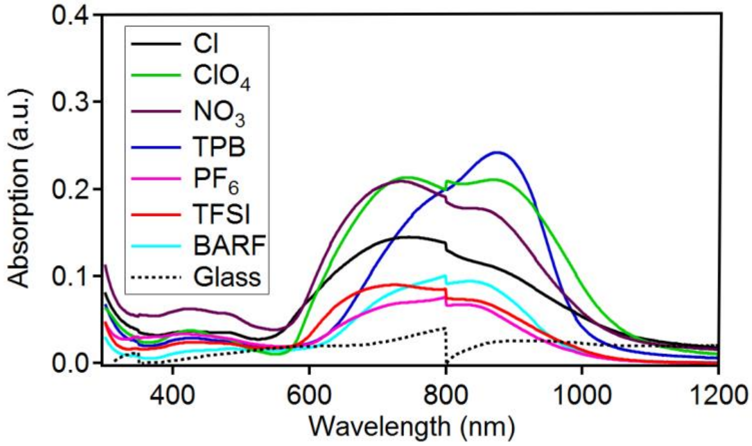

Figure S25. Raw film absorption spectrum not normalized. The blank glass absorption spectrum with no dye is shown for comparison.

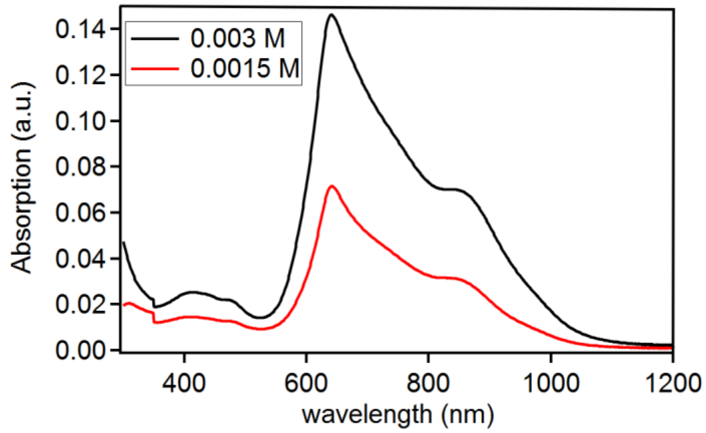

Figure S26. Film absorption with **C5-Cl** on glass prepared with varying concentrations of the dye.

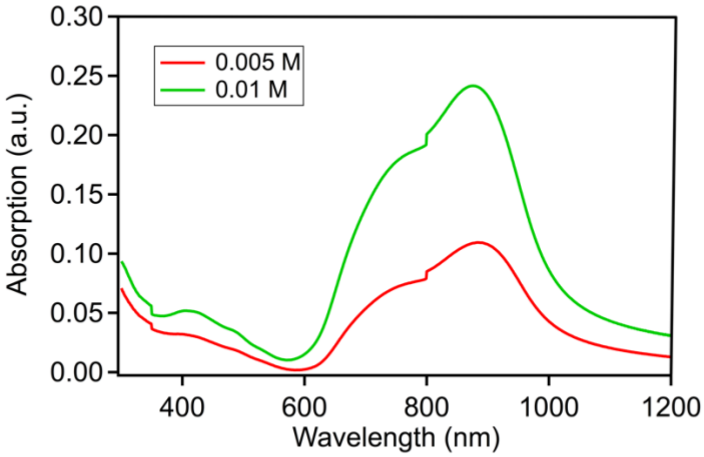

Figure S27. Film absorption with **C5-TPB** on glass prepared with varying concentrations of the dye.

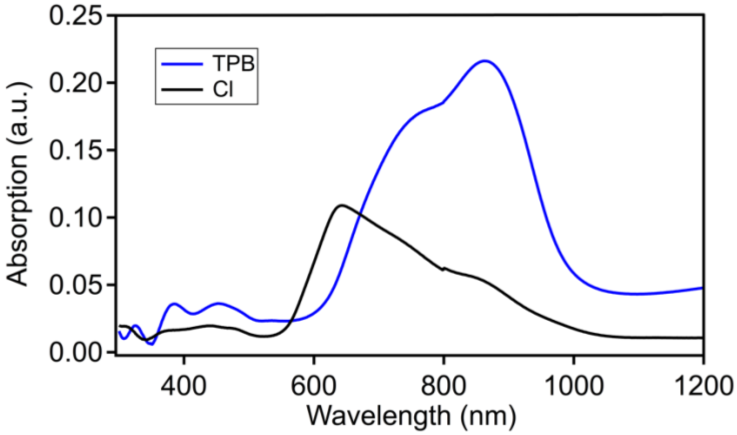

Figure S28. Film absorption with **C5-TPB** and **C5-Cl** on fluorine doped tin oxide (FTO).
